# Integration of Artificial Intelligence and Quantum Transport toward Stereoselective Identification of Carbohydrate Isomers

**DOI:** 10.1021/acscentsci.4c00630

**Published:** 2024-08-06

**Authors:** Sneha Mittal, Milan Kumar Jena, Biswarup Pathak

**Affiliations:** † Department of Chemistry, Indian Institute of Technology (IIT) Indore, Indore, Madhya Pradesh 453552, India

## Abstract

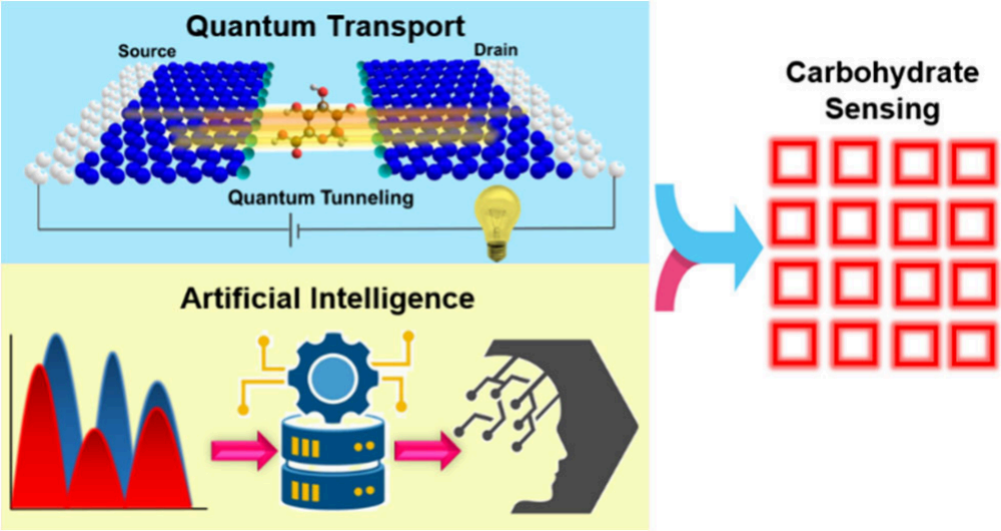

Detection of stereoisomers of carbohydrates with molecular resolution, a challenging goal analysts desire to achieve, is key to the full development of glycosciences. Despite the promise that analytical techniques made, including widely used nuclear magnetic resonance and mass spectrometry, high throughput *de novo* carbohydrate sequencing remains an unsolved issue. Notably, while next-generation sequencing technologies are readily available for DNA and proteins, they are conspicuously absent for carbohydrates due to the immense stereochemical and structural complexity inherent in these molecules. In this work, we report a novel computational technique that employs quantum tunneling coupled with artificial intelligence to detect complex carbohydrate anomers and stereoisomers with excellent sensitivity. The quantum tunneling footprints of carbohydrate isomers show high distinguishability with an in-depth analysis of underlying chemistry. Our findings open up a new route for carbohydrate sensing, which can be seamlessly integrated with next-generation sequencing technology for real-time analysis.

## Abstract 2

Integration of quantum tunneling with artificial intelligence achieves the sensing of complex carbohydrate isomers, both anomers and stereoisomers, at single molecule resolution with a maximum accuracy score of 100%.

## Introduction

Carbohydrates, mainly glycans, are essential to understand a broad range of biological processes such as cell signaling and adhesion,^1^ inflammation,^2^ protein folding,^3^ signal transduction,^4^ immune responses,^5^ and pathogen recognition.^6^ Additionally, glycans on eukaryotic cell surfaces are often associated with cancer and inflammation,^7^ while O-glycosylation can contribute to various neurodegenerative disorders like Parkinson’s,^8^ Alzheimer’s,^9^ and Huntington’s diseases.^10^ Distinguishing carbohydrate stereoisomers is crucial to unveil their biological roles and potential applications as vaccinating and therapeutic agents.^11−14^ Several analytical techniques have been developed so far for carbohydrate identification and characterization, including nuclear magnetic resonance spectroscopy, mass spectrometry, liquid chromatography, and ion mobility spectrometry, among others.^15−20^ However, owing to their vast structural diversity, pyranose and furanose ring sizes, stereochemistry, anomericity, and diverse regiochemistry of glycosidic linkages, single-molecular identification of carbohydrates could not be achieved until now.^21−23^ The development of a fast and cost-effective technology capable of accurately decoding the complex sugar code is an urgent and critical need of the hour.^24^ It is striking that theoretical investigations, which have been widely made for next-generation protein and DNA sequencing, have not yet been demonstrated for carbohydrates, representing a critical deficit in our understanding of biological systems.

**Figure 1:**
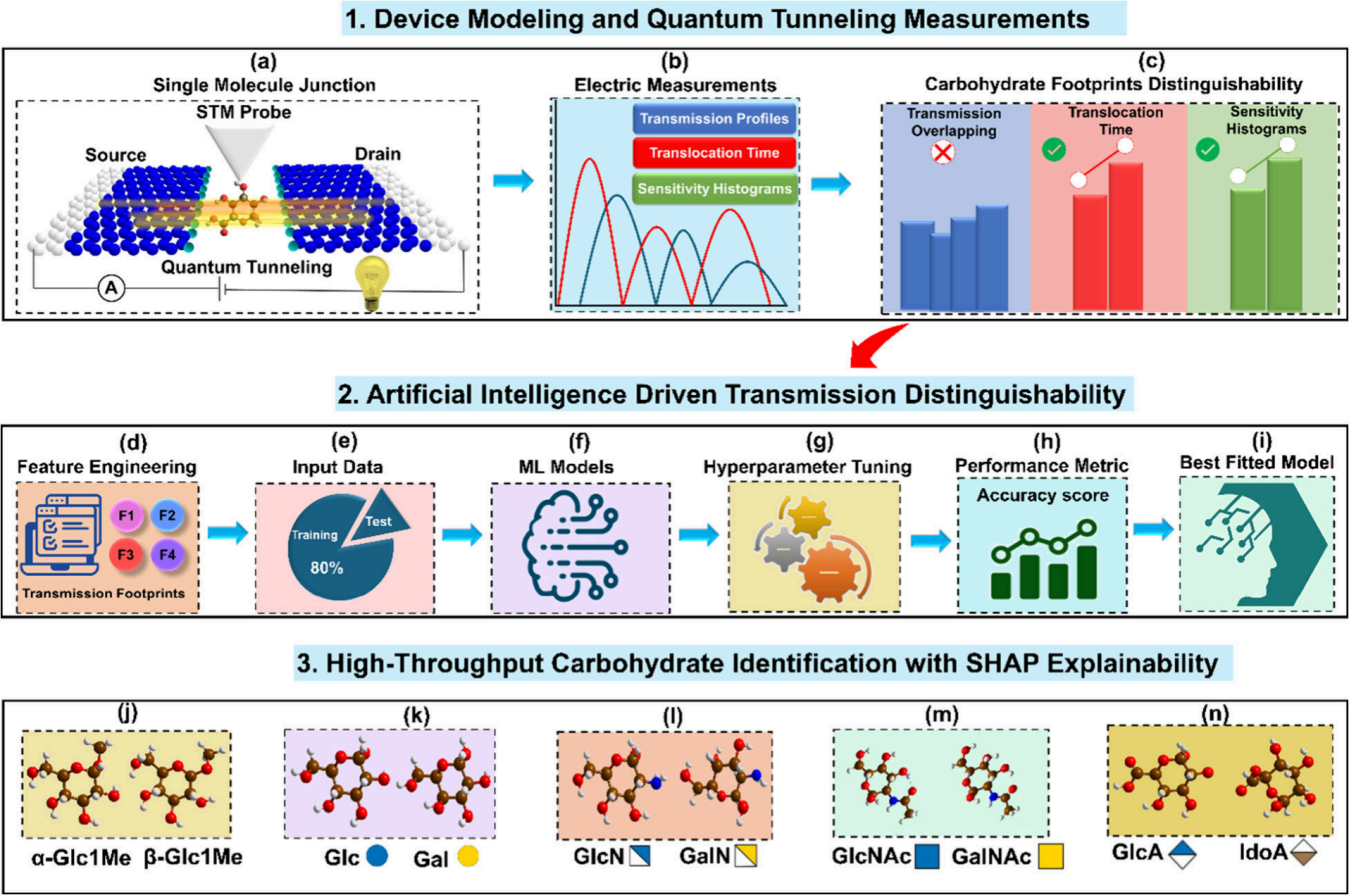
Overview of quantum tunneling coupled artificial intelligence workflow. (a) Schematic of single molecule graphene tunneling junction through which a wide variety of carbohydrate isomers can be trapped in all possible orientations (see Methods for details). The proposed tunneling junction is a field effect transistor-based device where the left and right electrodes act as the source (*V*_S_) and drain (*V*_D_) of electrons, respectively. When a gate voltage (*V*_G_) is applied, the tunneling of electrons generates characteristic tunneling signals, which can be measured by using the STM probe over the immobilized molecule, (b) electric fingerprints, including transmission-energy profiles, translocation time and sensitivity histograms used for identification of carbohydrate isomers, (c) carbohydrate footprints analysis for their distinguishability at single molecule level. We scrutinized all the electric footprints of carbohydrate isomers and encountered a significant challenge in decoding transmission profiles precisely into carbohydrate alphabets, which prompted us to explore AI tools for fast and accurate identification, (d) feature engineering to transform the raw transmission data into a set of informative features for training of machine learning (ML) model, (e) splitting of prepared transmission input data set, 80% used for training of the ML models and the rest 20% used for assessing their predictive performance on unseen data, (f) selection of ML models based on their predictive performance on test data set, (g) tuning of hyperparameters to enhance the efficiency of ML models toward unknown data prediction, (h) assessing the performance metrics, accuracy scores to choose the best-fitted model for carbohydrate classification, (i) utilization of best fitted model for prediction of class of carbohydrate isomers, (j) identification of α-Glc1Me and β-Glc1Me, (k) identification of Glc and Gal, (l) identification of amine derivatives, GlcN and GalN, (m) identification of GlcNAc and GalNAc, and (n) identification of acid derivatives, GlcA and IdoA.

Giving attention to a timely need for rapid and cheap high-throughput carbohydrate sensing, herein, we develop a new computational tool that couples the nanoscale quantum tunneling (QT) approach with artificial intelligence (AI) for the identification of a good number of carbohydrate isomers, both anomers and stereoisomers, at single molecule resolution (Figure 1). The proposed approach is capable of addressing the potential challenges of cumbersome analytical techniques such as large sample requirement, high time and cost, complex data interpretation, poor resolution between the closely related isomers, the inability to distinguish stereoisomers, and can perform ultrafast and high throughput carbohydrate sensing with an in-depth understanding of underlying chemistry.^25^

## Results and Discussion

### Quantum Transport

When a molecule is trapped in between the graphene nanogap electrodes, the QT of electrons generates characteristic tunneling signals, which can be measured by using the conducting tip of a STM probe over the immobilized molecule, providing a direct pathway to identify the individual molecule.^26^ The concept of QT is actively being utilized in sequencing genome and proteome and can be further extended to sequence carbohydrates.^27^ The idea is that carbohydrate isomers may have different coupling sites close to the electrode edges (owing to a change in the configuration), which may lead to a change in the local density of states and, subsequently, a change in the molecular orbital coupling between the carbohydrate and electrode. Any change in the electrode–molecule coupling is exponentially sensitive to the transmission signals.^28^ Therefore, we sought to calculate the transmission footprints of carbohydrates, which would allow us to identify each carbohydrate isomer individually.

To provide a comprehensive picture of carbohydrate sensing, we have considered a total of ten carbohydrate isomers, two anomeric isomers, namely, methyl α-d-glucopyranoside (α-Glc1Me) and methyl β-d-glucopyranoside (β-Glc1Me) and other carbohydrate stereoisomers, namely, α-d-Glucose (Glc) and α-d-Galactose (Gal); α-d-Glucosamine (GlcN) and α-d-Galactosamine (GalN); α-d-*N*-Acetyl-Glucosamine (GlcNAc) and α-d-*N*-Acetyl-Galactosamine (GalNAc); α-d-Glucuronic Acid (GlcA) and β-l-Iduronic acid (IdoA) in this study. The selection of these carbohydrates is driven by their pivotal roles in fundamental biological processes.^29,30^ Notably, glucose (Glc) and galactose (Gal) serve as major building blocks for human glycans, forming the structural foundation of essential biological molecules.^12^ Amino sugars, including Glucosamine (GlcN) and Galactosamine (GalN), play critical roles as components of glycoproteins and glycolipids, contributing significantly to cellular recognition and signaling mechanisms.^31^ While carbohydrate derivatives such as acidic (GlcA and IdoA) and amine sugars (GlcNAc and GalNAc) are key components of complex glycosaminoglycans and play an important role in various physiological and pathophysiological functions.^32^

To mimic the dynamics of carbohydrates inside the junction, both rotation and translation dynamics have been taken into consideration (Figure S1). Prior to QT calculations, each carbohydrate is relaxed inside the tunneling junction for the minimum energy configuration (Figure S2). Simulation results show that the 13.7 Å wide graphene nanogap tunneling junction is sufficient to accommodate each considered carbohydrate isomer in all possible orientations. It should be noted that the device gap size that we proposed for monosaccharides might not be suitable for the detection of disaccharides or oligosaccharides. In the case of oligosaccharides, both intramolecular and intermolecular hydrogen bondings may take place. These hydrogen bonding interactions may improve the molecular orbital coupling between the carbohydrate and electrode, resulting in a shift of molecular states with respect to the Fermi level. The robust electrode–molecule coupling may lead to alignment of the HOMO level with the electrochemical potential of the nanogap device, which may facilitate the resonant quantum transport. In resonant transport, the molecular energy levels carry the current, increasing by several orders of magnitude. We expect that in the case of oligosaccharides with improved hydrogen bonding interactions, resonant transport may occur, which can lead to the amplification of transmission signals. For their identification, the gap of the device needs to be tuned so that a wide variety of oligosaccharides, as well as monosaccharides with all possible hydrogen bonding interactions, can be accommodated over a dynamic configuration space.

**Figure 2:**
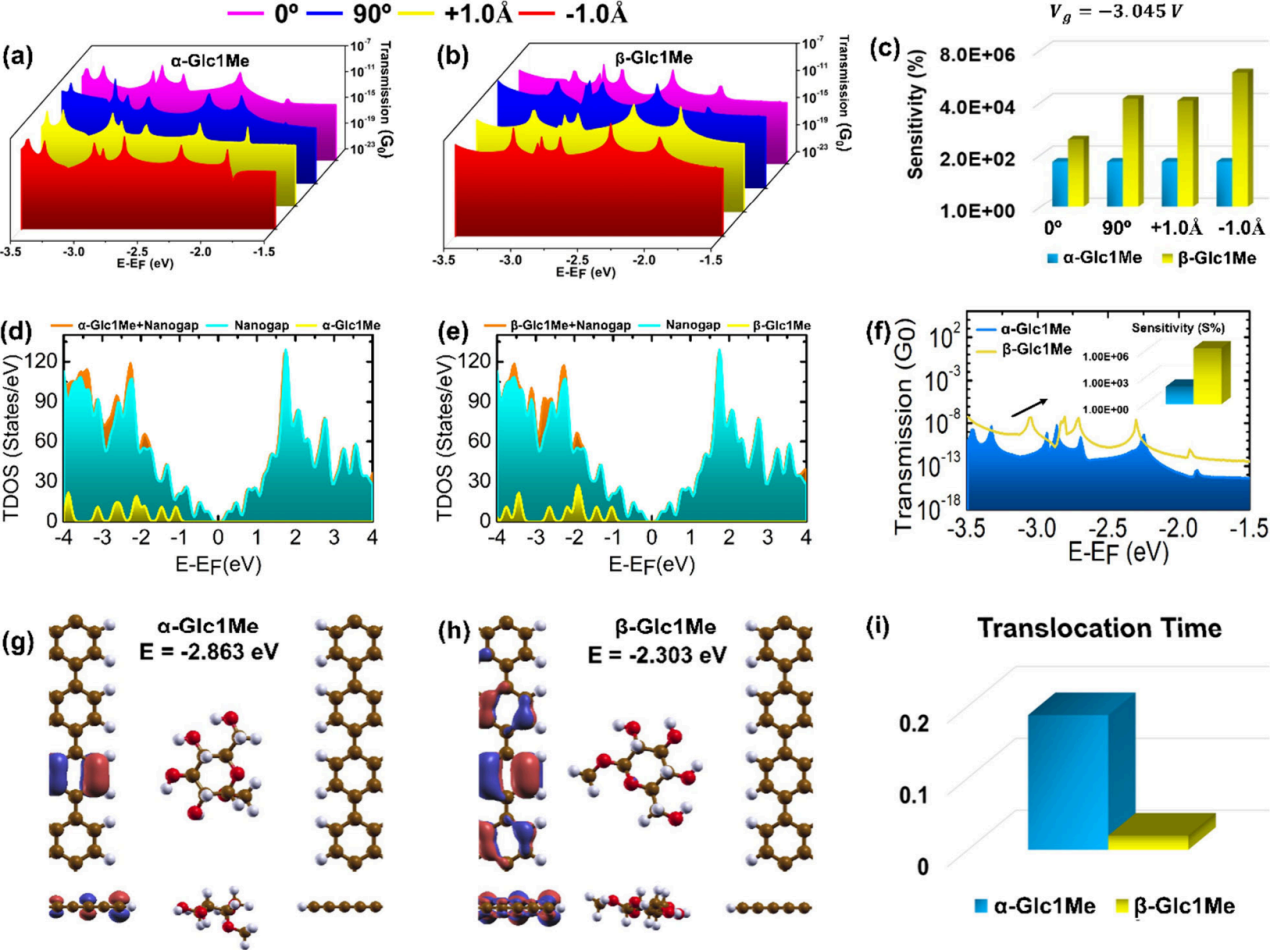
QT readouts of carbohydrate anomers α-Glc1Me and β-Glc1Me. (a) Zoomed zero-bias transmission energy profiles of α-Glc1Me in the energy window of −3.5 to −1.5 eV with different rotation and translation dynamics, and (b) zoomed zero-bias transmission energy profiles of β-Glc1Me in the energy window of −3.5 to −1.5 eV with different rotation and translation dynamics. The Fermi energy level is set to zero, with (c) sensitivity histograms at a gate voltage of −3.045 V illustrating significant distinguishability between the anomers with orientational variations. The transmission sensitivity *S*% = , where *G*_*x*_ and *G*_0_ are the conductance of individual carbohydrate and reference carbohydrate (with lower transmission value), respectively, (d) total density of states (TDOS) plot of α-Glc1Me while trapped inside the tunneling junction, (e) total density of states (TDOS) plot of β-Glc1Me while trapped inside the tunneling junction, (f) combined transmission energy profiles of α-Glc1Me and β-Glc1Me in their minimum energy configuration. Inset shows the corresponding sensitivity (S%) bar plot at energies as marked by arrow, (g) molecular orbitals (MOs) isosurface plot of α-Glc1Me at an energy value of −2.863 eV, (h) molecular orbitals (MOs) isosurface plot of β-Glc1Me at an energy value of −2.303 eV. In the MOs wave function plots (isosurface value is 0.01 e/Å^3^), the positive and negative lobes are shown in red and blue colors, respectively, (i) translocation time bar plot for α-Glc1Me and β-Glc1Me. As per Boltzmann’s relation (τ ∼ *e*^–*E*_*i*_^/*k*_B_*T*), translocation time (τ) is highly dependent on the electrode–molecule interaction energy (*E*_*i*_). Atom color code: C (brown), H (white), N (blue), and O (red).

The QT is first applied to carbohydrate anomers α-Glc1Me and β-Glc1Me to calculate their electric and molecular footprints while trapped inside the tunneling junction. These carbohydrates differed only in the relative orientation of the −OCH_3_ (methoxy) group. As shown in Figure 2a,b, transmission footprints have a significant correlation with the orientational variation, and a slight change in the orientation can lead to an exponential change in the transmission values, which may give rise to a large variation in the transmission signals for a single isomer. For a quantitative understanding of the device distinguishability between the anomers, sensitivity histograms are analyzed, which demonstrates that the carbohydrate anomers can be selectively identified at a particular energy value (Figure 2c).

To better understand the electronic picture of carbohydrate sensing, we further study the electronic density of states of graphene-carbohydrate-graphene systems. We find that the molecular states localized on isolated carbohydrates are exclusively present below the Fermi level and exhibit spatial orbital overlapping with states localized on graphene nanogap electrodes, resulting in significant molecular orbital coupling and better distinguishability below the Fermi level, particularly in the energy range of −3.5 to −1.5 eV (Figure 2d,e). Transmission footprints in the minimum energy configuration have also been analyzed with sensitivity histograms, which clearly show that the identification of carbohydrate anomers is possible through QT with excellent resolution (Figure 2f). To get physical insights into the electric footprints, molecular orbital (MOs) isosurface plots have been demonstrated, which show stronger electrode–molecule coupling in the case of β-Glc1Me, as reflected in the higher density of MOs, which leads to increased transmission (Figure 2g,h).

**Figure 3:**
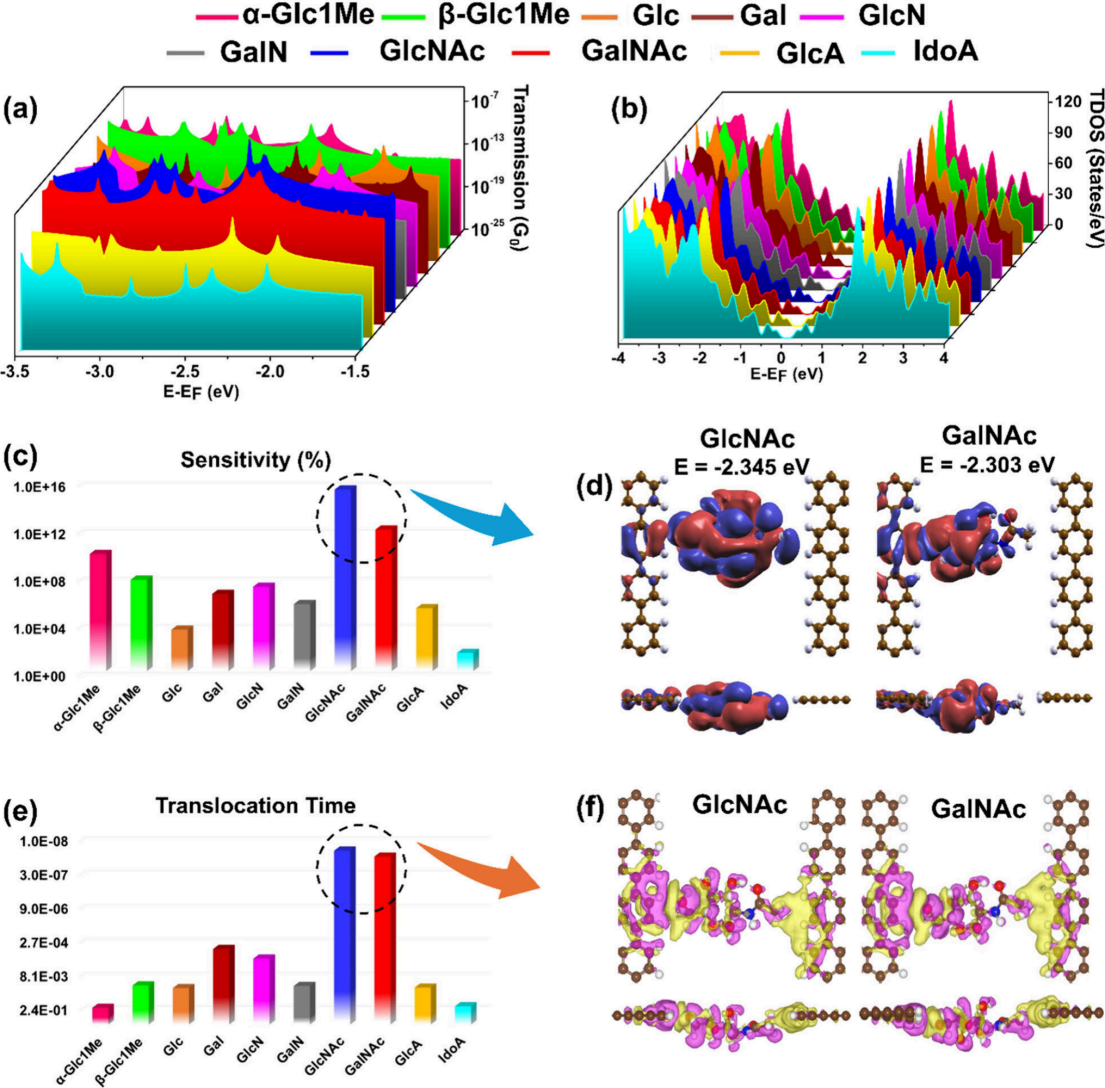
Signature footprints of individual carbohydrate isomers in their minimum energy configuration. (a) Transmission footprints in the energy window of −3.5 to −1.5 eV. The Fermi energy level is shifted to zero, (b) total density of states (TDOS) plots of each carbohydrate when trapped inside the tunneling junction, (c) sensitivity histograms for simultaneous sensing of all carbohydrate anomers and stereoisomers at a gate voltage of −2.863 V, (d) molecular orbitals isosurface plot of GlcNAc and GalNAc (isosurface value is 0.01 e/Å^3^). The positive and negative lobes are shown in red and blue colors, respectively, (e) molecular footprints for sensing of carbohydrate anomers and stereoisomers, and (f) charge density difference plots for GlcNAc and GalNAc. Atom color code: C (brown), H (white), N (blue), and O (red).

Apart from electric footprints, molecular footprint translocation time is also evaluated for anomers distinguishability, and results show that along with electric footprints molecular footprints can also be utilized for carbohydrate identification at high resolution (Figure 2i). To get a visual understanding of electrode-carbohydrate interaction, see charge density difference (CDD) plots (Figure S3), which demonstrate significant overlapping between the electron clouds of β-Glc1Me and electrode edges, leading to a relatively high interaction energy and low translocation time. The QT readouts of other carbohydrate stereoisomers are given in the Supporting Information (Figure S4–S8). For each carbohydrate stereoisomer, unique key electric and molecular footprints are observed, which consistently support the potential applicability of QT in high throughput carbohydrate sensing at single-molecule resolution. It is also crucial to have a look at the combined transmission profiles of all ten carbohydrate isomers because it will better provide the qualitative trend of real-time conductance data of carbohydrates being translocated through the junction in a steplike manner. As can be seen in Figure 3a, there is a significant degree of overlap between the transmission spikes, which may give rise to difficulty in decoding carbohydrate alphabets. From the density of states analysis, it is observed that for each isomer, the contribution of HOMO is more toward the distinct transmission spikes (Figure 3b).

The quantitative analysis of sensitivity values indicates that by applying a gate voltage of −2.863 V, sensing of both carbohydrate anomers and stereoisomers can be achieved simultaneously (Figure 3c). Herein, the reported values are just possible values of gate voltages that can lead to significant distinguishability between the stereoisomers. It may vary in real-time measurements. We have reported values below the Fermi level because of maximum distinguishability; other values close to the Fermi level can also be considered. Interestingly, in the case of GlcNAc and GalNAc, the sensitivity values are relatively high, which could be attributed to the high density of MOs wave functions indicating strong electrode–molecule orbital coupling leading to resonant charge transport and high transmission (Figure 3d). Apart from sensitivity histograms, the translocation time footprints of carbohydrates are also significant in their individual sensing. Using translocation time values, a large number of carbohydrate isomers can be identified simultaneously with good resolution. As in electric footprints, the unusual behavior of GlcNAc and GalNAc is also reflected in their molecular footprints (Figure 3e). This unusuality in molecular footprints is a consequence of the high degree of overlap between the electron clouds of carbohydrates and electrode edges, causing a high charge transfer and strong interaction between the electrode and carbohydrates (Figure 3f). As a result of this strong interaction between the carbohydrates and electrodes, high transmission values and low translocation time are observed for GlcNAc and GalNAc. To get an overview of the transmission profiles of carbohydrates and their sensitivity histograms in different rotation and translation dynamics, see Supporting Information (Figure S9).

From the discussion so far, it is evident that the electric and molecular footprints of carbohydrates are promising for their distinguishability with different orientational variations. However, significant overlap between the transmission profiles of carbohydrate isomers might create a hurdle in signal interpretation and classification at the single-molecule level. This issue demands an advanced tool that can be easily integrated with the tunneling junction and can decode each carbohydrate isomer accurately from their transmission readouts. AI has the ability to be readily integrated with next-generation sequencing (NGS) devices and decode biomolecules with high precision and at significantly reduced time and cost.^33−37^ Given the vital significance of AI in achieving rapid and high-throughput biosensing, our next step involved harnessing AI to decode QT signals into carbohydrate alphabets.

### Integration of Artificial Intelligence

To avoid any type of overfitting and underfitting in the prediction, training the machine with well-sampled data is the foremost requirement. Hence, to call carbohydrate isomers through AI, we have first prepared a well-sampled input training data set. In a realistic analysis, we will have only the electric signal readouts without any preceding information about the carbohydrate sequence. Hence, the identification of carbohydrates is performed by extracting the features directly from their transmission readouts (see Methods for details). Next, we split the input training into three parts: train (80%), test (10%), and validation (10%) data sets. Subsequently, we have employed six machine learning (ML) classification algorithms available at the open-source scikit-learn library,^38^ namely, random forest classification (RFC), logistic regression (LR), k-nearest neighbors classification (KNC), decision tree classification (DTC), support vector machines (SVM), and feedforward neural network (FNN) and calculated the accuracy score for prediction of test and validation data sets. To increase the accuracy, these algorithms are utilized at their best hyperparameters. (For details, see Table S1). The computed test and validation accuracy scores with optimized ML algorithms are listed in Table S2. Looking at Table S2, the best-fitted algorithm for the classification of carbohydrate isomer pairs is random forest classification (RFC), with classification accuracy >95% for each isomer pair.

**Figure 4:**
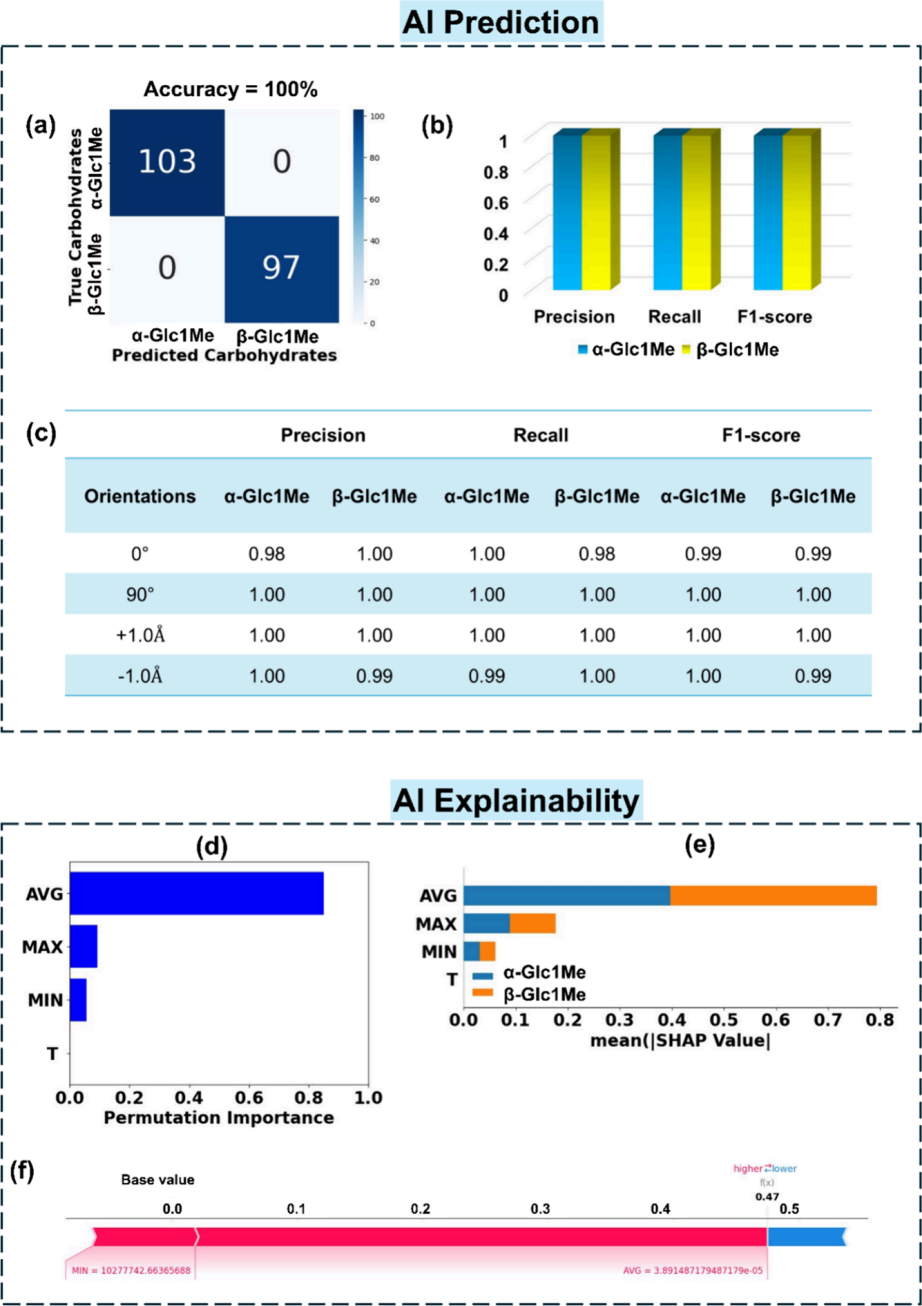
AI prediction of carbohydrate anomers α-Glc1Me and β-Glc1Me with explainability. (a) Confusion matrix for RFC prediction in their minimum energy configuration inside the tunneling junction, (b) classification report enclosing parameters precision, recall, and f1-score, (c) performance metrics for RFC prediction with different rotation and translation dynamics, (d) global feature importance plot, (e) SHAP summary bar plot illustrating the contribution of each feature toward individual class, and (f) SHAP summary force plot illustrating the contribution of features toward single prediction.

The best-fit RFC is first utilized for the classification of carbohydrate anomers α-Glc1Me and β-Glc1Me. As shown in Figure 4a,b, the RFC algorithm is able to call each anomer in their most likely configuration with a perfect classification accuracy of 100%. The algorithm also performed consistently well in the prediction of the class of anomers with both rotation and translation dynamics, as reflected in their quite good classification report enclosing parameters precision, recall, and f1-score in the range of 0.98–1.00 (Figure 4c).

To enhance the explainability of the RFC prediction, we analyze the global feature importance plot, as given in Figure 4d. We notice that the prediction is mainly driven by the feature AVG, which could be attributed to the significant change in the scaling relationship between the features and output when average normalized transmission values are used. To further understand the contribution of each feature toward the ML prediction, Shapley additive explanations (SHAP) are made. SHAP is a cooperative game theory-based technique that estimates the significance of each feature within a model and provides a consistent and interpretable method for comprehending the predictions made by ML models at both global and local levels.^39^ It helps in elucidating the complex relationship between the features and ML descriptors driving the machine’s decision-making process. Features with positive SHAP values positively impact the prediction, while those with negative values have a negative impact. A better understanding of how each feature value affects the prediction of a single carbohydrate can be found in the analyzed SHAP (Shapley Additive exPlanations) summary bar plot (Figure 4e). The height of the bars shows that in calling α-MeGlu, the contribution of the AVG feature is significantly high. To understand how a single prediction is influenced by the extracted features, SHAP force plots are demonstrated, which reveals that in this single prediction features AVG and MIN are pushing the prediction toward a higher value, while the rest of the other features are pushing toward a lower value (Figure 4f) and as a result of this features interplay, the output value 0.47 is predicted. After the successful implication of RFC in calling carbohydrate anomers, we further utilized it to call other carbohydrate stereoisomers (see Supporting Information and Figure S10–S13 for RFC prediction of carbohydrate stereoisomers). The AI calling results of each carbohydrate anomer and stereoisomer pair with different rotation and translation dynamics are summarized in Table 1.

**Table 1:**
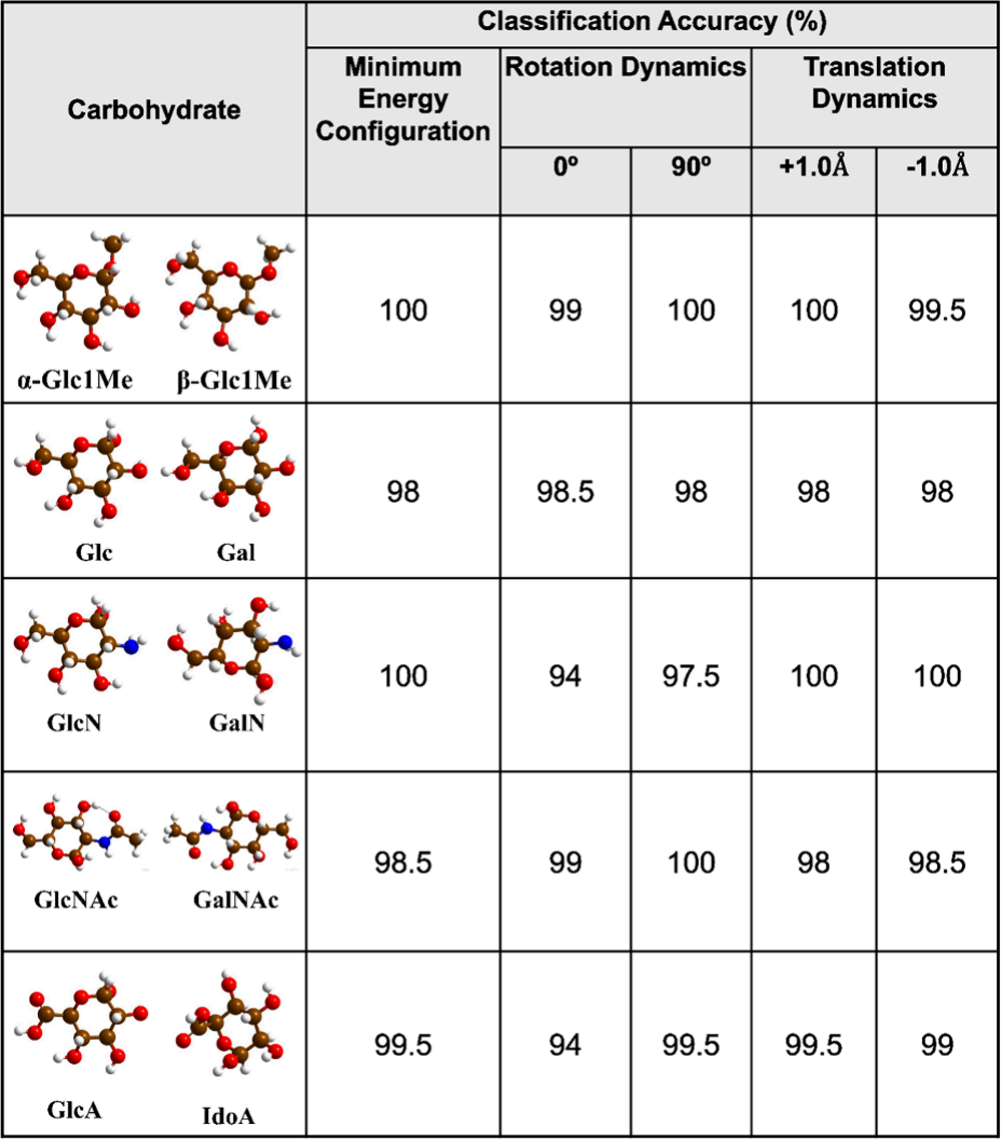
Classification Accuracy for Carbohydrate Isomers Identification by RFC

As shown in Table 1, the RFC algorithm has been able to distinguish carbohydrates α-Glc1Me, GlcN, and GlcNAc from their respective counterparts with a maximum accuracy score of 100%, and other isomers Glc-Gal and GlcA-IdoA are classified with a maximum accuracy of 98.5% and 99.5%, respectively. Note that under real-time conditions, these carbohydrates may adopt any orientation with respect to the tunneling gap, and the calculated results indicate the potential of RFC in distinguishing the carbohydrate anomer and epimer pairs in different orientations with high precision and accuracy. To ensure the generalizability and stability of the RFC algorithm in calling carbohydrates, a 10-fold cross-validation is employed. The performed 10-fold cross-validation is a robust validation method to check whether the data is overfitted or not.^40−43^ The method involves dividing data into 10 equal parts or “folds” leveraging each fold iteratively for both training and validation. The model is trained 10 times, with each iteration using 9-fold for training and the remaining 1-fold for the validation. This procedure is repeated until each fold has been utilized once as the validation data set. This ensures its effectiveness across a wide range of diverse data sets. As can be seen in Table 2, in each case of carbohydrate isomer pair, the average accuracy score of 10 folds of cross-validation is close to the test accuracy score, which suggests that the RFC algorithm is perfectly stable and there is no overfitting or underfitting in the predictions.

**Table 2:**
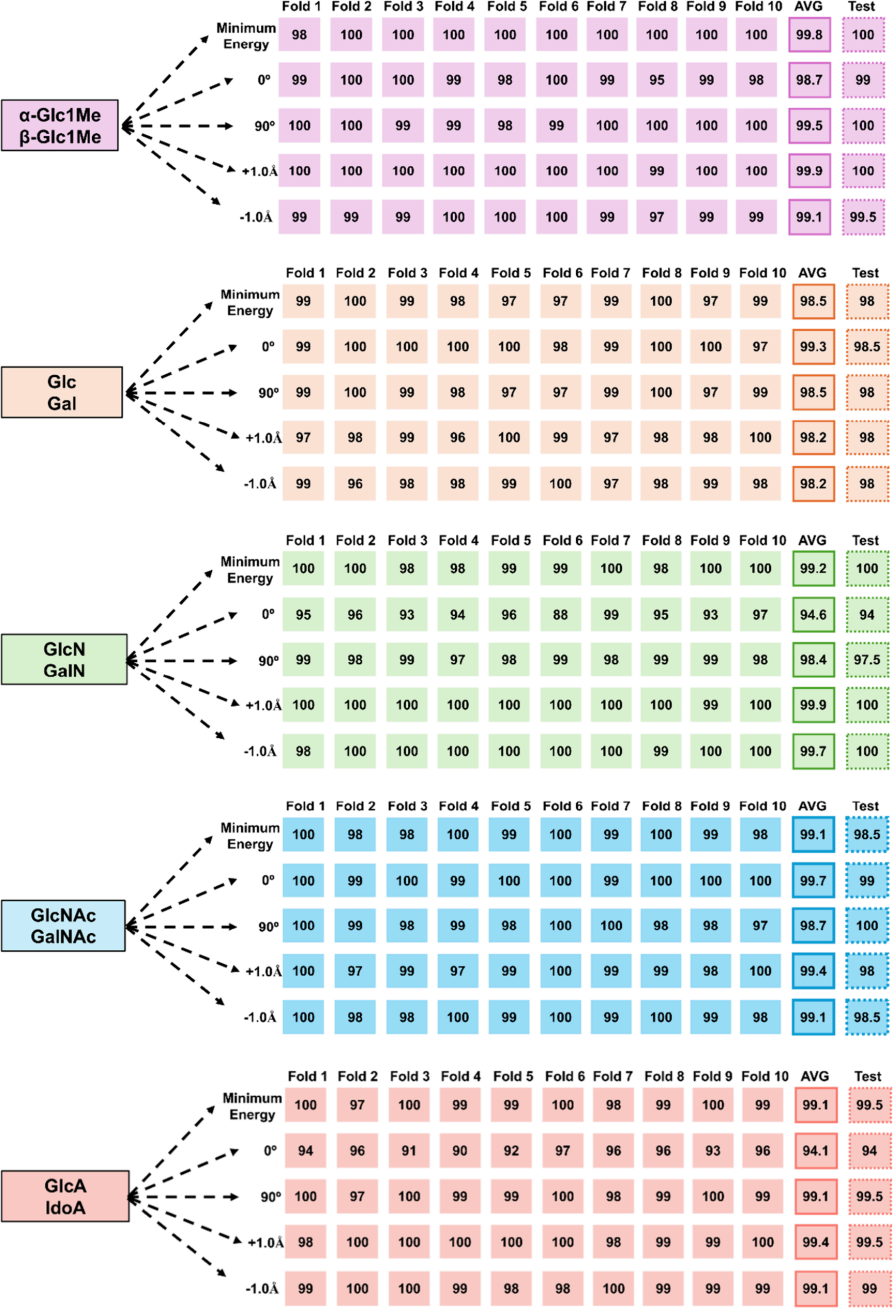
10-Fold Cross Validation for Carbohydrate Isomers Identification by RFC

Here, it would be important to see how the descriptors influence the prediction of carbohydrate isomer pairs. For different isomer pairs, we observed different feature contribution hierarchies (Table 3).

**Table 3:**
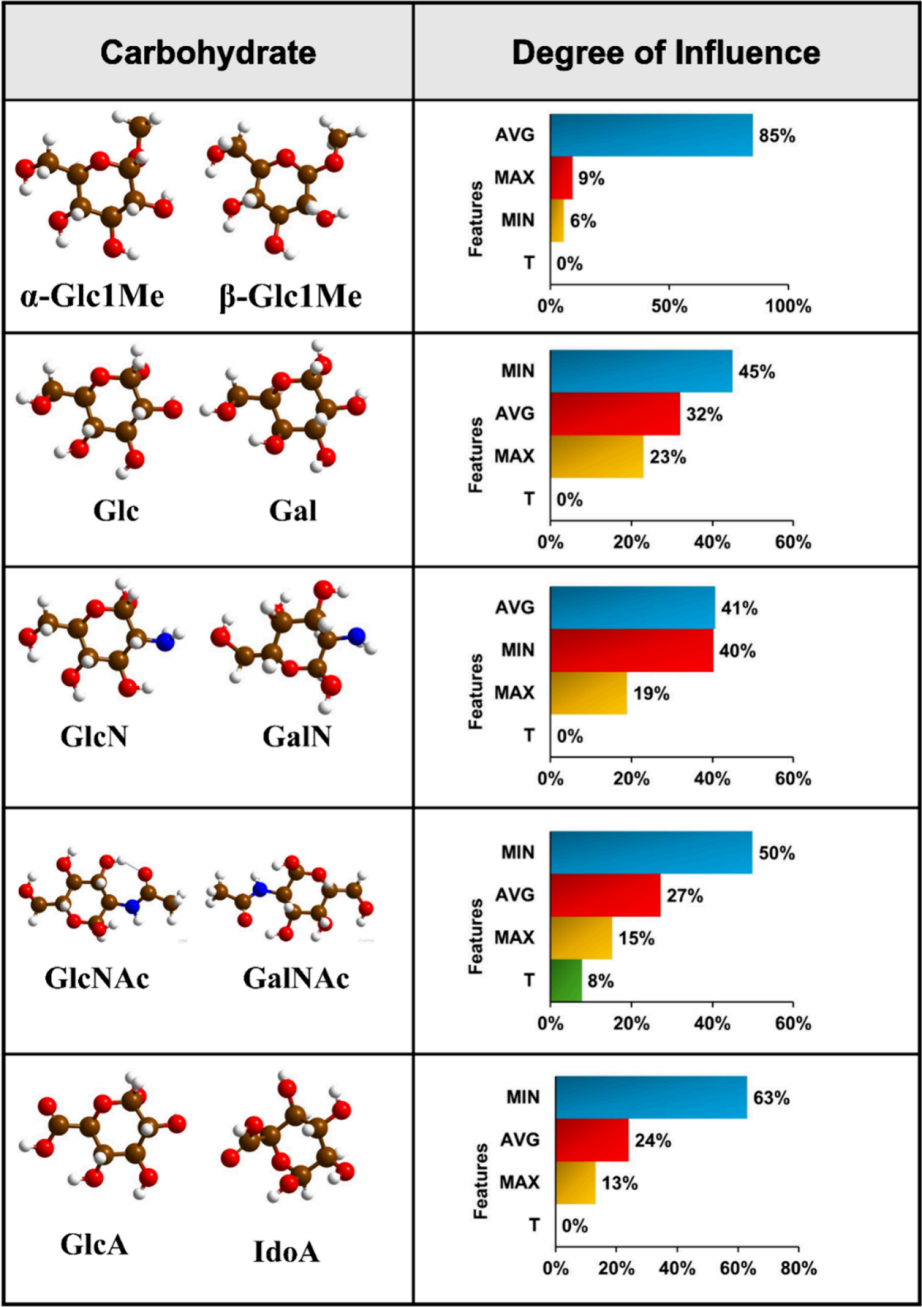
Degree of Feature Influence for Carbohydrate Isomer Pair Identification

Majorly in the predictions, the most influential features are MIN and AVG. Their high contribution could be attributed to the significantly changed scaling relationship when the transmission feature values are normalized by the minimum and average values, respectively. Here, we find that feature T has zero contribution to the predictions of carbohydrate isomer pairs except GlcNAc-GalNAc. Like electric and molecular footprints, the unusual behavior of the carbohydrate isomer pair GlcNAc-GalNAc is also reflected in their AI predictions. Compared to other carbohydrate isomers, GlcNAc and GalNAc exhibit strong electrode–molecule orbital coupling, leading to resonant charge transport and high transmission values, and this could be the reason that feature T has a certain contribution (8%) toward the prediction.

**Figure 5:**
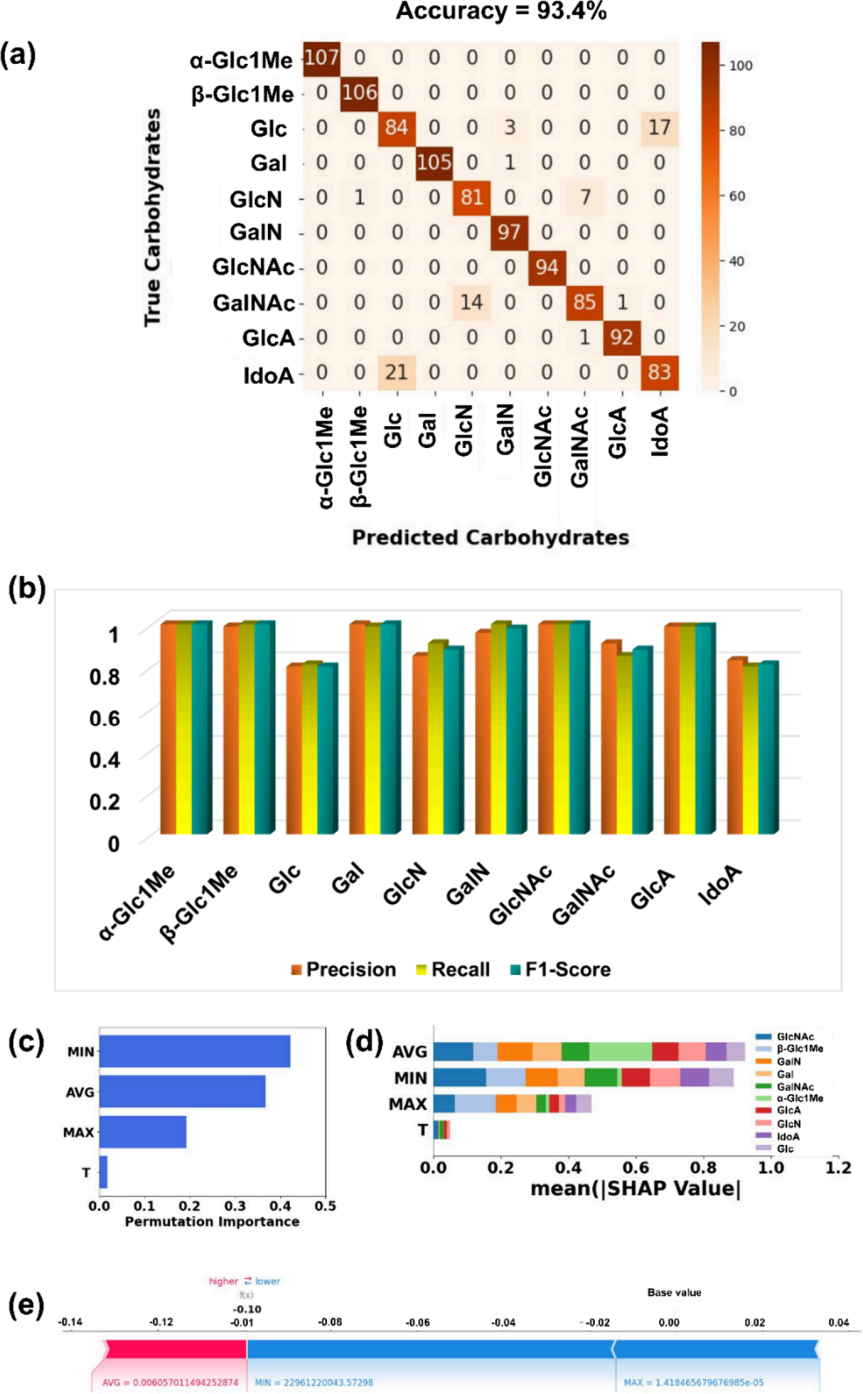
AI prediction of individual carbohydrates from a pool of QT transmission signals of all carbohydrate isomers. (a) Confusion matrix for identification of individual carbohydrates in their minimum energy configuration inside the tunneling junction, (b) classification report enclosing performance metrics precision, recall, and f1-score, (c) global feature importance plot, (d) SHAP summary bar plot illustrating the contribution of each feature toward prediction of individual carbohydrate class, and (e) SHAP summary force plot illustrating the contribution of features toward single prediction.

Compared with relative anomer and epimer identification, individual identification of carbohydrate isomers would be more difficult. In an attempt to achieve that, we extend the potential of RFC by calling each individual isomer separately from a pool of transmission data of all carbohydrate isomers. As shown in Figure 5a, RFC predicted each individual analyte with a pretty good accuracy score of 93.4% when trained with a complex data set of all 10 carbohydrates simultaneously. Even when trained with complex and overlapped data having similar transmission values, RFC still can call α-Glc1Me, Gal, and GlcNAc with a perfect accuracy of 100%. Here, the algorithm is mainly confused in calling the isomers Glc, GlcN, GalNAc, and IdoA. The potential reasons behind these miscalling events of RFC algorithms could be the structural, configurational, and functional group similarity of these carbohydrate isomers and the close resemblance in their transmission profiles leading to data sets with similar data points. Notably, both GlcN and GalNAc are amine derivatives of carbohydrate stereoisomers, while IdoA is the C-5 epimer of Glc, and this may cause difficulties in their identification at high resolution. The classification report is also found to be good, as reflected in the parameters: precision, recall, and f1-score, which are close to 1 (Figure 5b).

To introduce an explainable RFC, feature importance plots and SHAP plots are also evaluated. The results show that prediction is mainly driven by the features MIN and AVG (Figure 5c), and in the accurate calling of α-Glc1Me, Gal, and GlcNAc, the features AVG and MIN are dominating (Figure 5d). The SHAP force plot results show that in the single prediction, the feature AVG is pushing the prediction toward a higher value, and the rest are pushing toward a lower value; the prediction is mainly driven by the feature MIN (Figure 5e).

To further check the potential of RFC in calling individual isomers, transmission profiles of different rotation and translation dynamics are also analyzed (see Supporting Information and Figure S14–S17). The RFC calling results of individual carbohydrates in different orientations are summarized in Table 4.

**Table 4:**
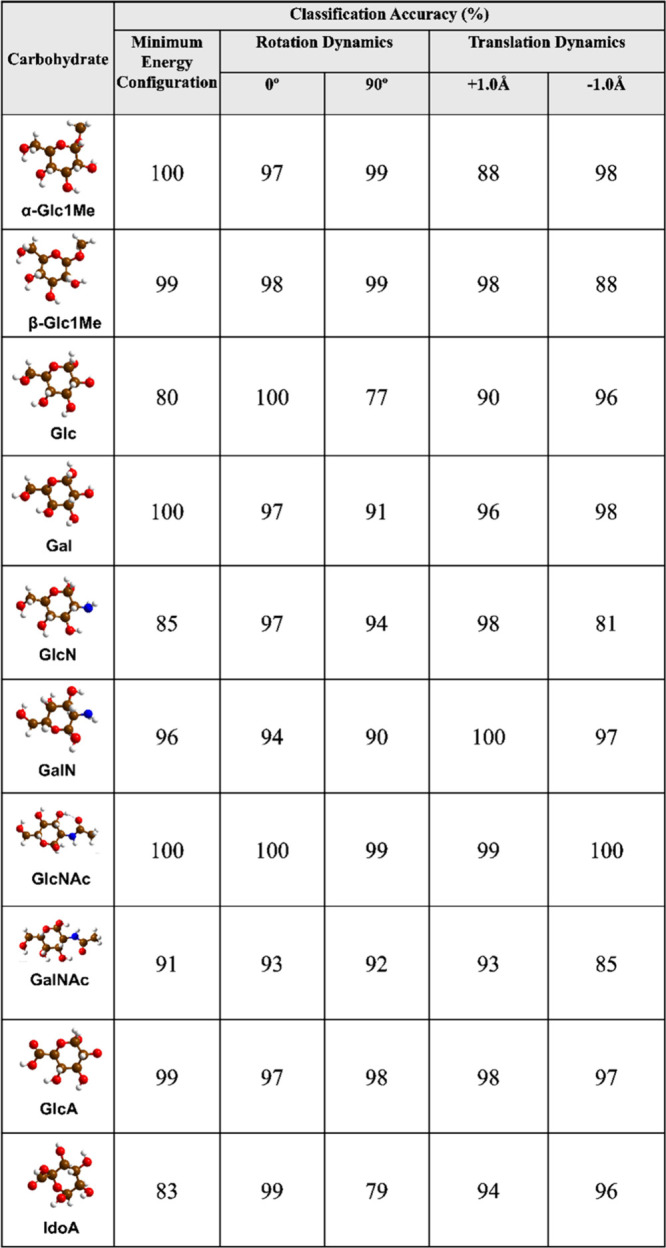
Classification Accuracy for Individual Carbohydrate Isomer Identification by RFC

The results show that when trained with transmission data of all carbohydrates collectively, RFC managed to call carbohydrates α-Glc1Me, Glc, Gal, GalN, and GlcNAc with a maximum accuracy of 100% and others β-Glc1Me, GluN, GalNAc, GluA, and IdoA with a maximum accuracy of 99%, 98%, 93%, 99%, and 99%, respectively. We also check the model stability in each prediction with 10-fold cross-validation, and the results show that along with isomer pair identification the RFC algorithm is well-suitable and generalized for individual carbohydrate prediction and not overfitted (Table S3).

To provide a generalized overview of the degree of feature influence in the prediction of individual carbohydrate isomers in their minimum energy configuration, we have summarized the feature contribution toward single monosaccharide prediction (Table 5).

**Table 5:**
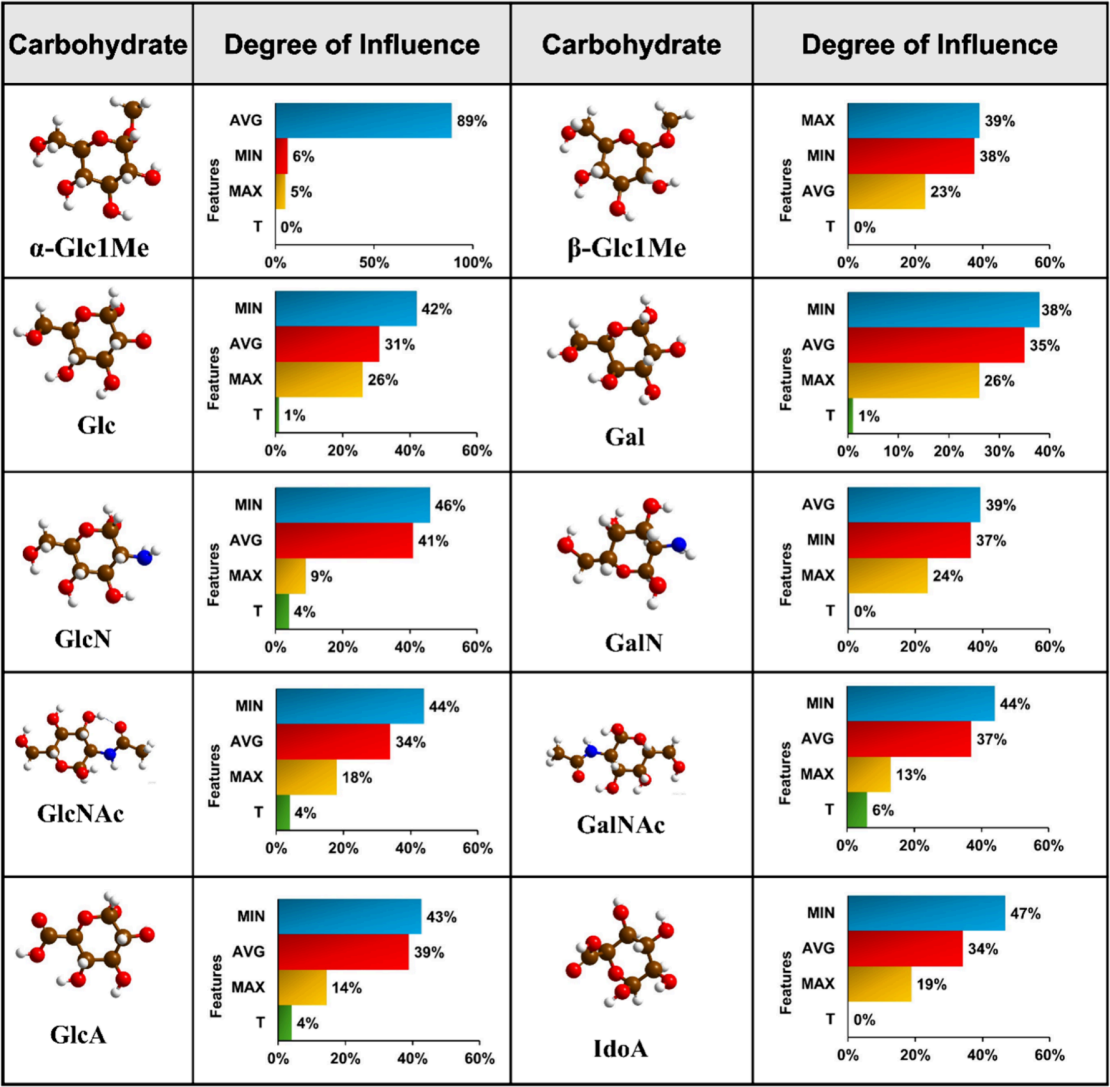
Degree of Feature Influence for Individual Identification of Monosaccharides

In the RFC prediction of anomers (α-Glc1Me and β-Glc1Me) as individual entities and within isomer pairs, feature T has zero contribution. Notably, in the case of stereoisomers with a Glc moiety (Glc, GlcN, GlcNAc, GlcA), the feature contribution hierarchy is noted to be the same. Carbohydrate derivatives such as acidic (GlcA and IdoA) and amine sugars (GlcNAc and GalNAc) were also found to share the same feature contribution hierarchy. In both the classification of carbohydrate stereoisomers as individual entities and within isomer pairs, the AVG and MIN feature notably exhibit greater influence, while the T feature contributes constantly less. This behavior is attributed to variations in the coupling strength between graphene electrodes and carbohydrate molecular orbitals (MOs). The division of transmission values by their average, minimum, and maximum counterparts induces a substantial shift in the scaling relationship among feature values, particularly when dealing with the overlapped transmission signals of carbohydrate stereoisomers. This transformative process significantly enhances the distinguishability. Conversely, employing actual transmission values (T), characterized by a notable degree of overlap, results in lesser distinguishability among carbohydrate stereoisomers.

## Conclusion

In summary, a computational technique is demonstrated for direct sensing of complex carbohydrate isomers with quantum tunneling (QT) coupled with artificial intelligence (AI). Different from the previously used methods, using a graphene nanogap tunneling junction, high throughput sensing of both carbohydrate anomers and stereoisomers is achieved with superior resolution. A total of ten carbohydrate isomers, which are the major building blocks of human glycans, are simultaneously identified with high sensitivity and selectivity. For a better understanding of QT readouts, an in-depth analysis of electrode–molecule coupling and electronic density of states is rendered. To check the real-time viability, the effect of both rotation and translation dynamics on QT signals is investigated, and a significant effect of orientational variations is noticed. To enhance signal interpretation and classification, an explainable artificial intelligence (AI) tool is developed, which can identify the carbohydrate isomers exclusively from their complex transmission readouts with a maximum sensing accuracy of 100%. Different from the analytical techniques, the technique is rapid and needs only a subpicomole sample of analyte to identify a wide variety of carbohydrate isomers, suggesting its potential application in glycomics and therapeutic decision-making.^25^ Since the nanopore technology has no limit on the number of molecules being translocated, if integrated with nanopore technology, an unlimited number of carbohydrates can be sequenced at a time using just a miniaturized portable device.

## Methods

### Single-Molecule Junction

To model the single-molecule junction, we first relaxed the graphene electrodes and proceeded to construct the nanogap tunneling junction by employing the first-principles density functional theory (DFT) calculations, as implemented in the SIESTA package.^44^ A gap of 13.7 Å, capable of accommodating all possible orientations of carbohydrate isomers and sufficient screening of the perturbation effect from the carbohydrates, is created between the electrodes in the zigzag direction. The dangling bonds of graphene nanogap edges are stabilized by hydrogen atoms, which are demonstrated to provide chemical contrast in STM images and sliding contacts to individual molecule through weak hydrogen bonding interactions.^45,46^

### Quantum Tunneling

Before QT calculations, the carbohydrate isomers are optimized separately by using the B3LYP/6-31+G* level of theory, as implemented in the Gaussian09 code,^47^ and then optimized again within the graphene nanogap using the DFT calculations, as implemented in the SIESTA code. To further check the initial geometries of considered monosaccharides, we have used other computational methods, such as wb97xd/def2tzvp and PBE0+D3/6-311+G(d,p), and found that the structures (with some minor differences) are also local minimum energy structures (Table S4). The minimum energy structures and their relative energy values are very similar to those obtained at the B3LYP/6-31+G* level of theory. To measure the QT readouts of graphene-carbohydrate-graphene systems, we have employed the nonequilibrium Green’s function (NEGF) based DFT calculations as implemented in the TranSIESTA package.^48^ For optimization, Troullier–Martins norm-conserved pseudopotentials,^49^ consistent-exchange van der Waals density functionals (vdW),^50^ and a double-ζ-polarized basis set (DZP) are used. To further check the optimized geometry of graphene-carbohydrate-graphene systems, we have optimized the systems again using a triple-ζ-polarized basis set (TZP) with additional polarization functions as available in SIESTA. At the higher level of calculations (vdW-TZP), the geometries of graphene-carbohydrate-graphene systems and relative energy values are very similar to those obtained at the vdW-DZP level of theory (Table S5). These results indicate that with other computational methods, the geometries of isolated carbohydrates and graphene-carbohydrate-graphene systems are very much identical. For all atom relaxation, a conjugate gradient (CG) algorithm with a 200 Ry cutoff is used. The transmission energy profiles *T*(*E*,*V*_*b*_) are calculated by using the equation,^51^
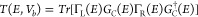
where, *G*_*C*_(*E*) and *G*_*C*_^†^(*E*) represent the retarded and advanced Green’s functions while Γ_L_(*E*) and Γ_R_(*E*) stand for the coupling matrices of the left and right electrodes, respectively.

Translocation time (τ) is calculated by using the Boltzmann’s relation (τ ∼ *e*^–*E*_*i*_^/*k*_B_*T*), where *k*_B_ is Boltzmann constant and *T* = 298 K. Interaction energy (*E*_*i*_) values are calculated by using the following equation:^52^
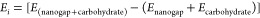


Here, *E*_(nanogap+carbohydrate)_ shows the total optimized energy of the graphene-carbohydrate-graphene system, *E*_nanogap_ and *E*_carbohydrate_ are the single-point energy values of isolated graphene nanogap and isolated carbohydrate in the minimum energy configuration of the graphene-carbohydrate-graphene system, respectively.

For the analysis of charge density difference [Δρ(*r*)] plots, the following equation is used,
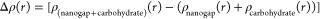


Here, ρ_(nanogap+carbohydrate)_ (*r*) shows the total charge density of the graphene-carbohydrate-graphene system and *ρ*_nanogap_(*r*) and *ρ*_carbohydrate_(*r*) are the charge density on the isolated graphene nanogap and isolated carbohydrate molecule in the minimum energy configuration of the graphene-carbohydrate-graphene system, respectively.

### Implementation of Artificial Intelligence

#### Input Data Sets

For the development of a robust ML method capable of identifying carbohydrate anomers and stereoisomers across dynamic configurations, we prepared input data sets for each considered five configurations: one minimum energy configuration, two rotation dynamics (0° and 90°), and two translation dynamics (+1.0 and −1.0 Å). For each carbohydrate isomer pair, this resulted in five distinct input data sets. To this end, for the identification of five carbohydrate isomer pairs (α-Glc1Me and β-Glc1Me, Glc and Gal, GlcN and GalN, GlcNAc and GalNAc, and GlcA and IdoA), we generated a total of 25 data sets (5 isomer pairs ×5 configurations = 25 data sets). Additionally, we performed the classification of individual monosaccharides (α-Glc1Me, β-Glc1Me, Glc, Gal, GlcN, GalN, GlcNAc, GalNAc, GlcA, and IdoA) across the same five configurations. For the classification of individual monosaccharides for each considered five configurations, we have a total of 5 data sets (1 data set for all ten monosaccharides ×5 configurations = 5 data sets). Therefore, the total number of data sets prepared for the robust classification of carbohydrate anomers and stereoisomers is 30 (25 data sets+ 5 data sets = 30 data sets). For each carbohydrate isomer, there are 500 transmission data points in an energy range of −3.5 to +3.5 eV, which lead to a total of 1000 (a pair of isomers) and 5000 transmission (for all 10 monosaccharides) data points in each prepared input data set for carbohydrate isomer pairs and individual monosaccharides, respectively.

#### Feature Generation

In real-time measurements, one has access to only conductance or transmission signals without any prior knowledge of the carbohydrate sequence. Therefore, herein, we primarily focus on the extraction of features directly from the transmission readouts. In a report by Farimani et al., the longitudinal ionic blockade current approach is used for the identification of amino acids.^53^ In their approach to machine-learning-based prediction, they used normalized (by the maximum value) and denormalized values of ionic current and residence time as features. Our approach to the stereoselective identification of carbohydrate isomers is based on the transverse current method. Different from the ionic current and residence time features, herein, we use transmission as a descriptor. Being inspired by the work and based on our domain knowledge in the field, herein, for the classification of carbohydrate isomers, we have considered both normalized (by the maximum, minimum, and average value) and denormalized values of transmission as features for our classification data sets. Thus, a total of four features have been extracted from the transmission energy profiles, namely, transmission (*T*), average normalized transmission (AVG), i.e.,  where *T*_avg_ is the average value of transmission, minima normalized transmission (MIN), i.e.,  where *T*_min_ is the minimum value of transmission, and maxima normalized transmission (MAX), i.e.,  where *T*_max_ is the maximum value of transmission.

### Performance Metrics

To analyze the performance of ML algorithms, we evaluated four performance metrics:
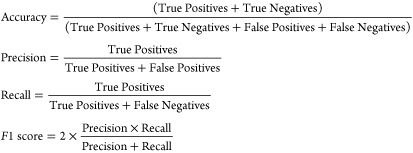


True Positives represent the number of instances correctly predicted as positive, True Negatives represent the number of instances correctly predicted as negative, False Positives represent the number of instances incorrectly predicted as positive, and False Negatives represent the number of instances incorrectly predicted as negative.

### Data Analysis

Corresponding to a single carbohydrate isomer, we have a total of 500 transmission data points in an energy range of −3.5 to +3.5 eV. Moreover, as we considered different spatial arrangements inside the graphene nanogap, corresponding to five orientations (0°, 90°, +1.0 Å, −1.0 Å, minimum energy configuration), we ended up with a total of 2,500 data points for a single isomer (500 data points for each configuration). To this end, a total of 25,000 transmission data points are analyzed for ten carbohydrate isomers with different spatial arrangements.

In training the ML models, 80% of the input training data set is used. Each input data set has a total of 1000 transmission values corresponding to two carbohydrate isomer pairs (500 for each isomer) and a total of 5000 transmission values corresponding to the 10 carbohydrate isomers in the energy range of −3.5 to +3.5 eV.

## Data Availability

The utilized machine learning models are available at Scikit-learn package 0.23.1, running in Python version 3.9.^38^ To construct, train, and run the codes, we used Google Colab 1.0.0.^54^ The data for reproducing the results are available upon reasonable request to the corresponding author.

## Supporting Information Available

Rotation and Translation Dynamics, Minimum Energy Configuration, Physical Insight into Molecular Footprints of carbohydrate Anomers, Transmission Energy profiles of Carbohydrate Stereoisomers, Sensitivity Histograms of Carbohydrate Stereoisomers, Density of States (DOS) of Carbohydrate Stereoisomers, Transmission of Carbohydrate Stereoisomers with Molecular Orbital Wave functions, Molecular Footprints of Carbohydrate Stereoisomers with Physical Insights, Transmission profiles and Sensitivity with Different Rotation and Translation Dynamics, Details of Optimized Hyperparameters, Test and Validation Accuracy Scores for Optimized ML Algorithms, RFC calling of Glc and Gal, RFC calling of GlcN and GalN, RFC calling of GlcNAc and GalNAc, RFC calling of GlcA and IdoA, RFC calling of All Ten Carbohydrate Isomers in 0° Orientation, RFC calling of All Ten Carbohydrate Isomers in 90° Orientation, RFC calling of All Ten Carbohydrate Isomers in +1.0 Å Orientation, RFC calling of All Ten Carbohydrate Isomers in −1.0 Å Orientation, 10-Fold Cross Validation, Optimized Geometries of Carbohydrates, Optimized Geometries of Graphene-Carbohydrate-Graphene Systems (DOCX)
