# Supplementary material for: Integration of Artificial Intelligence and Quantum Transport toward Stereoselective Identification of Carbohydrate Isomers
Source: ACS Cent Sci. 2024 Aug 6;10(9):1689–702. doi: 10.1021/acscentsci.4c00630 (PMC11428302; doi:10.1021/acscentsci.4c00630)
Supplement: Supplementary file 1 — oc4c00630_si_001.docx [file oc4c00630_si_001.docx]

***Supporting Information***

**Integration of Artificial Intelligence and Quantum Transport Towards Stereoselective Identification of Carbohydrate Isomers**

Sneha Mittal, ^†^ Milan Kumar Jena, ^†^ Biswarup Pathak*^, †^

^†^Department of Chemistry, Indian Institute of Technology (IIT) Indore, Indore, Madhya Pradesh, 453552, India

*E-mail: [biswarup@iiti.ac.in](mailto:biswarup@iiti.ac.in)

**1. Rotation and Translation Dynamics**

To mimic the dynamics of carbohydrates inside the junction, a total of four orientations, two in-plane rotations, and two out-of-plane translations have been taken into consideration. To address the effect of **rotation dynamics,** we have considered a possible rotation from 0° to 90° around the x-axis in the yz-plane for each considered carbohydrate inside the tunneling junction as shown in **Figure S1a**, while to mimic the **translational dynamics**, we have translated the carbohydrate isomer in both upward (+1.0 Å) and downward (-1.0 Å) directions from the initial position (0.0 Å) along the x-axis in the yz-plane as shown in **Figure S1b.**

**
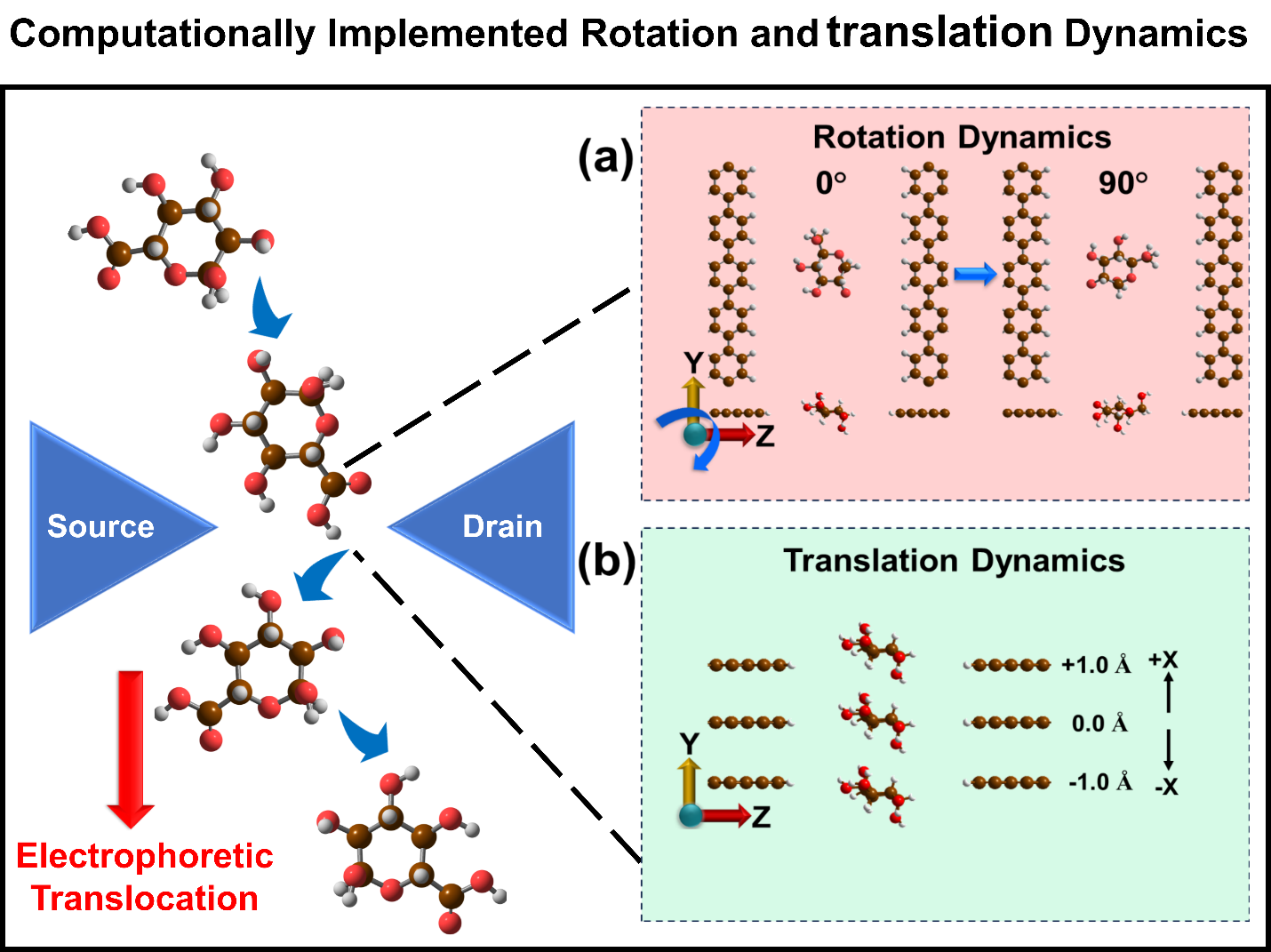
**

**Figure S1:** Schematic of carbohydrate electrophoretic translocation event inside the single molecule junction, representing significant orientational variations under real-time conditions. To mimic the dynamics of carbohydrates inside the junction, both in-plane rotation and out-of-plane translation dynamics have been considered, **(a)** representative orientations of Glc carbohydrate inside the single-molecule tunneling junction illustrated corresponding to rotation from 0° to 90° around the x-axis in the yz-plane and **(b)** representation of Glc carbohydrate translated out-of-plane along the x-axis in the yz-plane, in both positive and negative directions by ± 1.0 Å.

**2. Minimum Energy Configuration**

To obtain the minimum energy configuration of carbohydrates inside the tunneling junction, we first optimize each carbohydrate inside the graphene nanogap under each considered orientation (0°, 90°, +1.0 Å, and -1.0 Å) and then proceeded to calculate the relative energy values. Based on the relative energy values, as shown in **Figure S2**, we determine the minimum energy configuration.

**
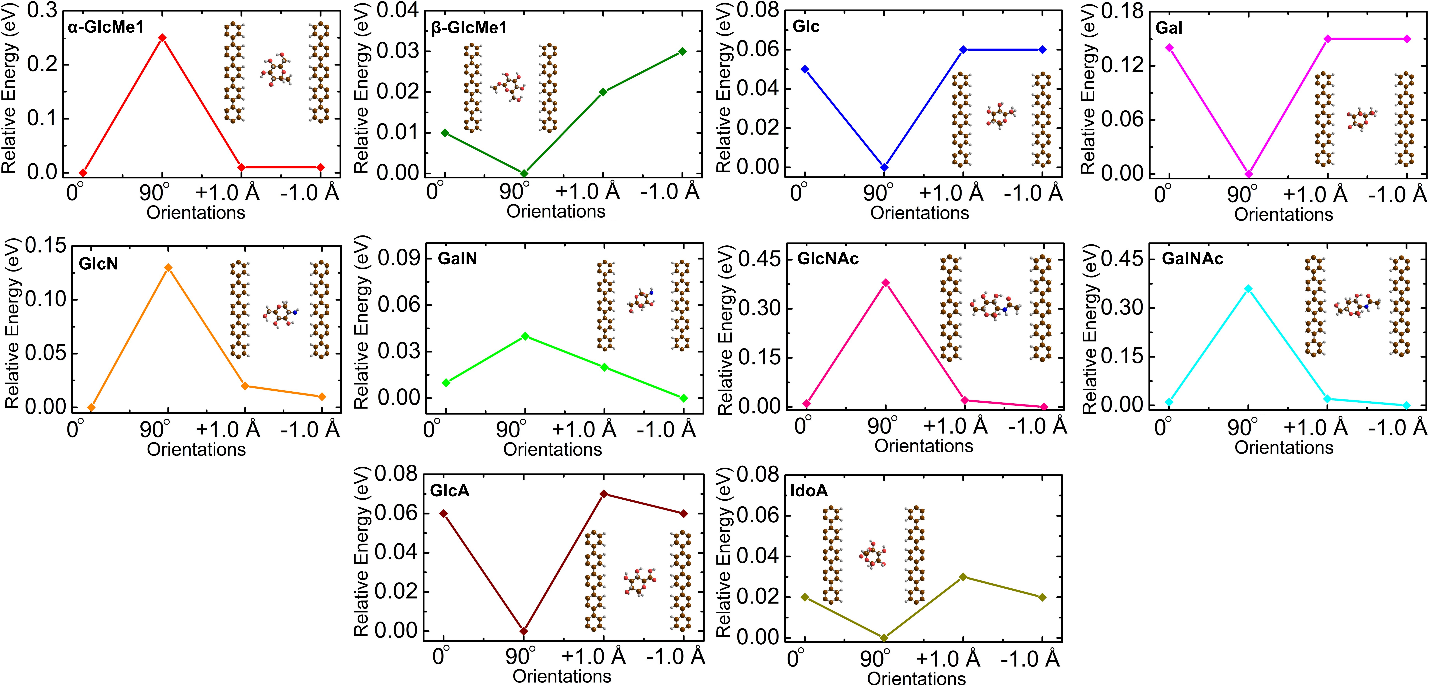
**

**Figure S2.** Relative energies (in eV) vs. orientations (0°, 90°, +1.0 Å, and -1.0 Å) plots for each carbohydrate molecule while relaxed between the nanogap electrodes. The most stable geometry of different graphene-carbohydrate tunneling junctions is shown in their respective insets. Atom color code: C (brown), H (white), N (blue), and O (red).

**3. Physical Insight into Molecular Fingerprints of carbohydrate Anomers**

**
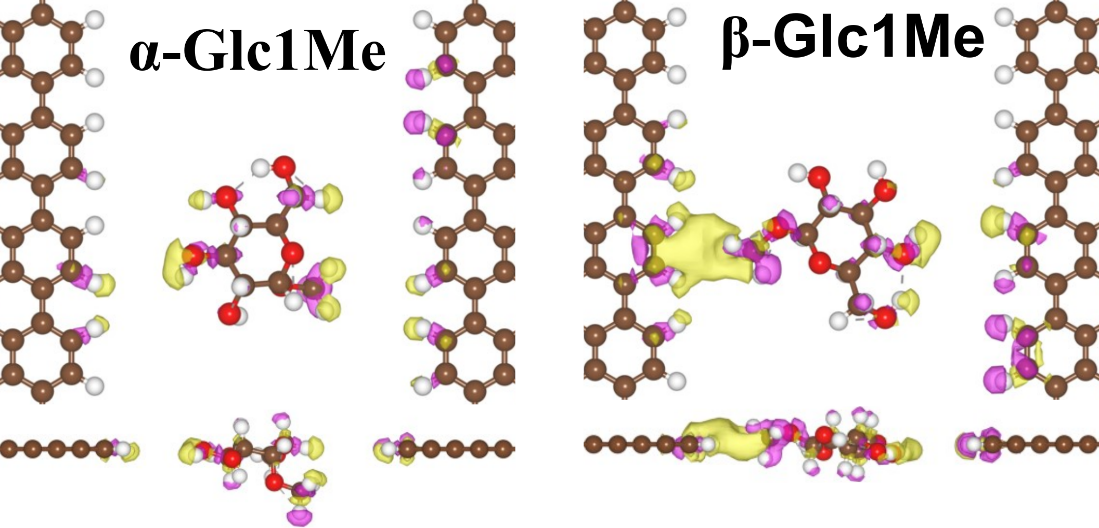
**

**Figure S3.** Charge density difference (CDD) plots (isosurface value is 0.0005 e/$Å^{3}$) for carbohydrate anomers α-Glc1Me and β-Glc1Me. The charge depletion and accumulation are represented by yellow and magenta colors, respectively. Atom color code: C (brown), H (white), N (blue), and O (red).

**4. Transmission Energy profiles of Carbohydrate Stereoisomers**


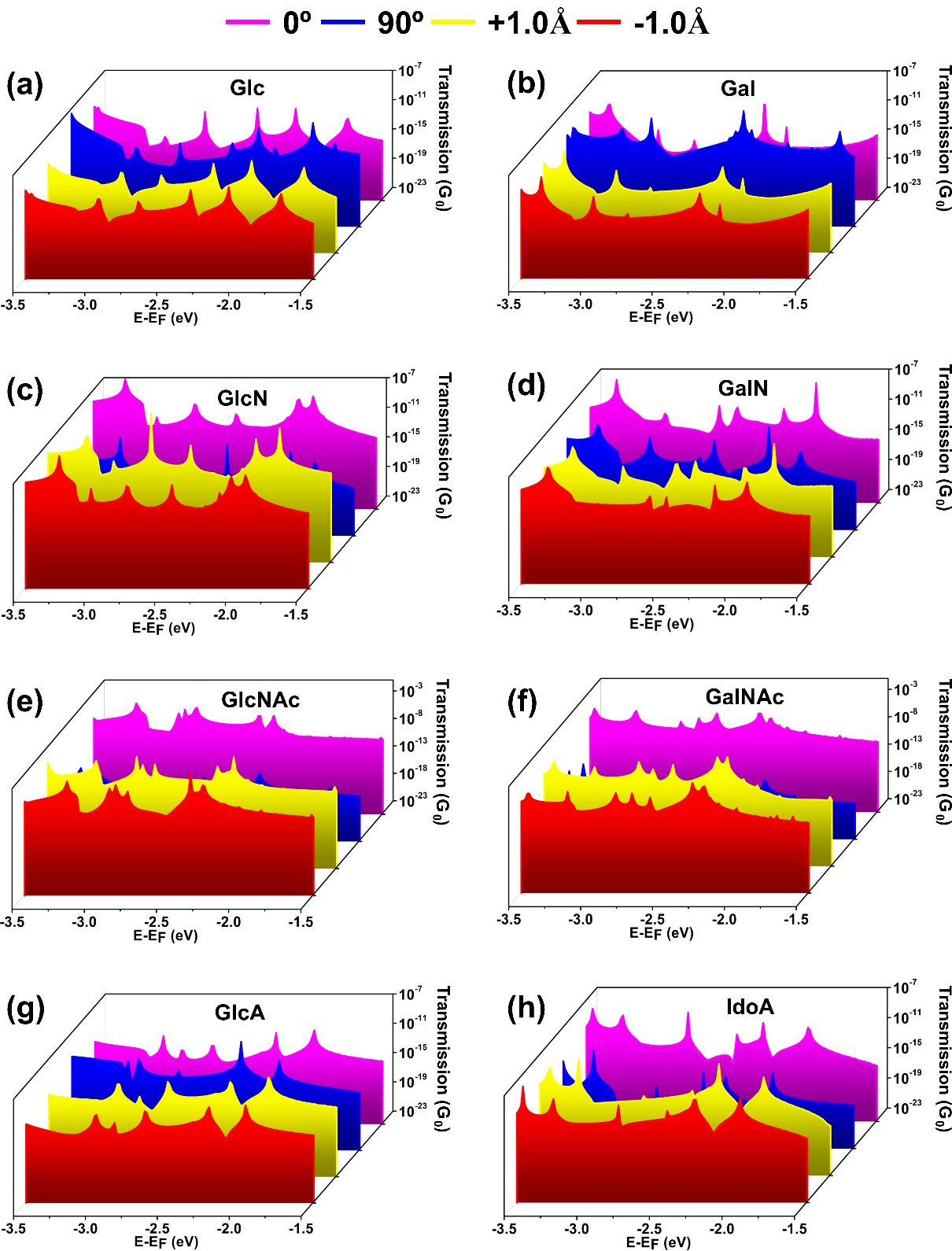


**Figure S4.** Zoomed zero-bias transmission energy profiles of carbohydrate stereoisomers in the energy window of -3.5 to -1.5 eV with different rotation and translation dynamics. **(a)** Glc and Gal, **(b)** GlcN and GalN, **(c)** GlcNAc and GalNAc, and **(d)** GlcA and IdoA. The Fermi energy level is shifted to zero.

**5. Sensitivity Histograms of Carbohydrate Stereoisomers**


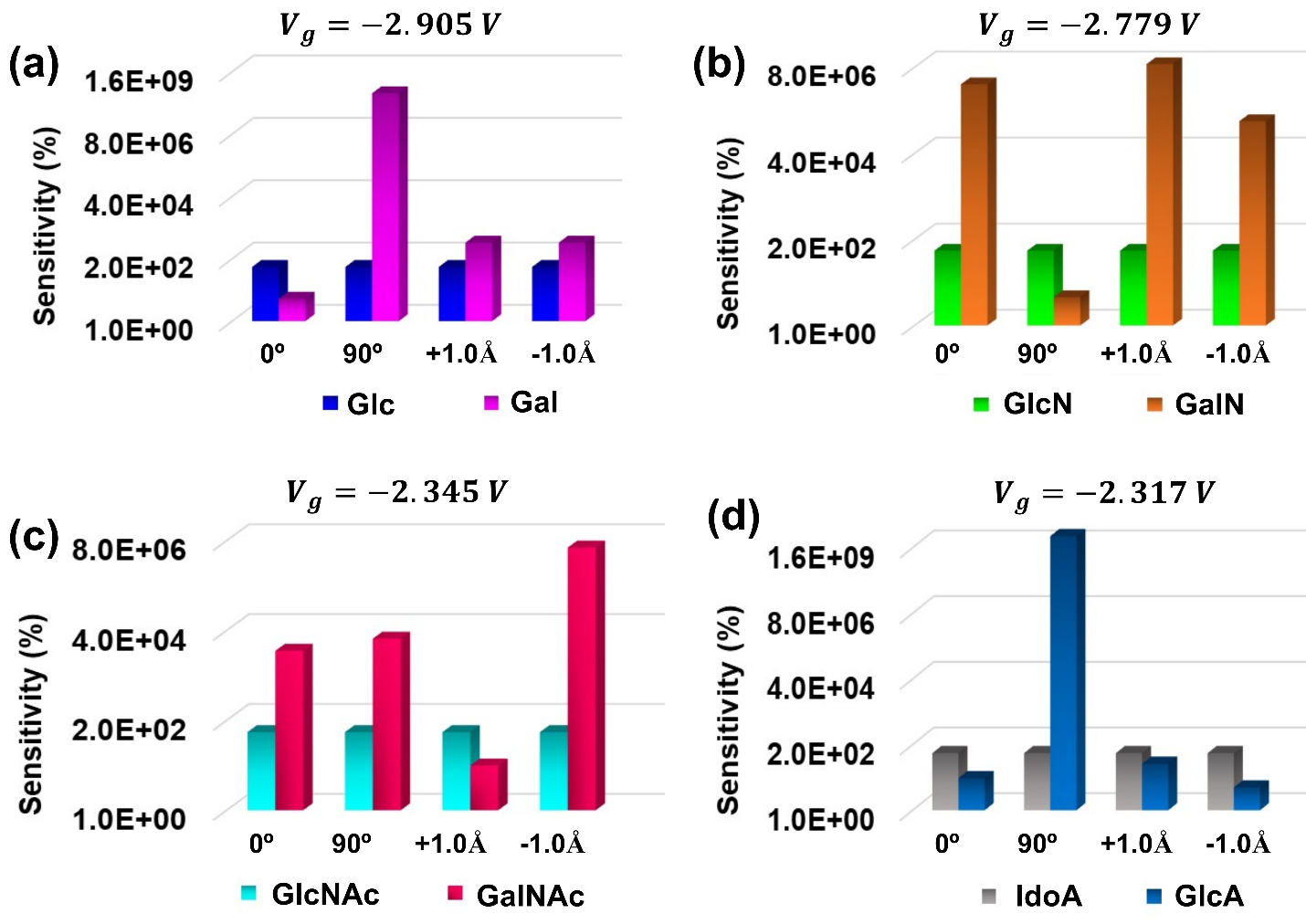


**Figure S5.** Sensitivity histogram bar plots for graphene-carbohydrate-graphene systems with different rotation and translation dynamics. **a)** Glc and Gal, **(b)** GlcN and GalN, **(c)** GlcNAc and GalNAc, and **(d)** GlcA and IdoA. Sensitivity (S%) = $\frac{G_{x}}{G_{0}}\times100$, where $G_{x}$ and $G_{0}$ are the conductance of carbohydrate isomers and reference carbohydrate isomer (with lower transmission value), respectively.

**6. Density of States (DOS) of Carbohydrate Stereoisomers**


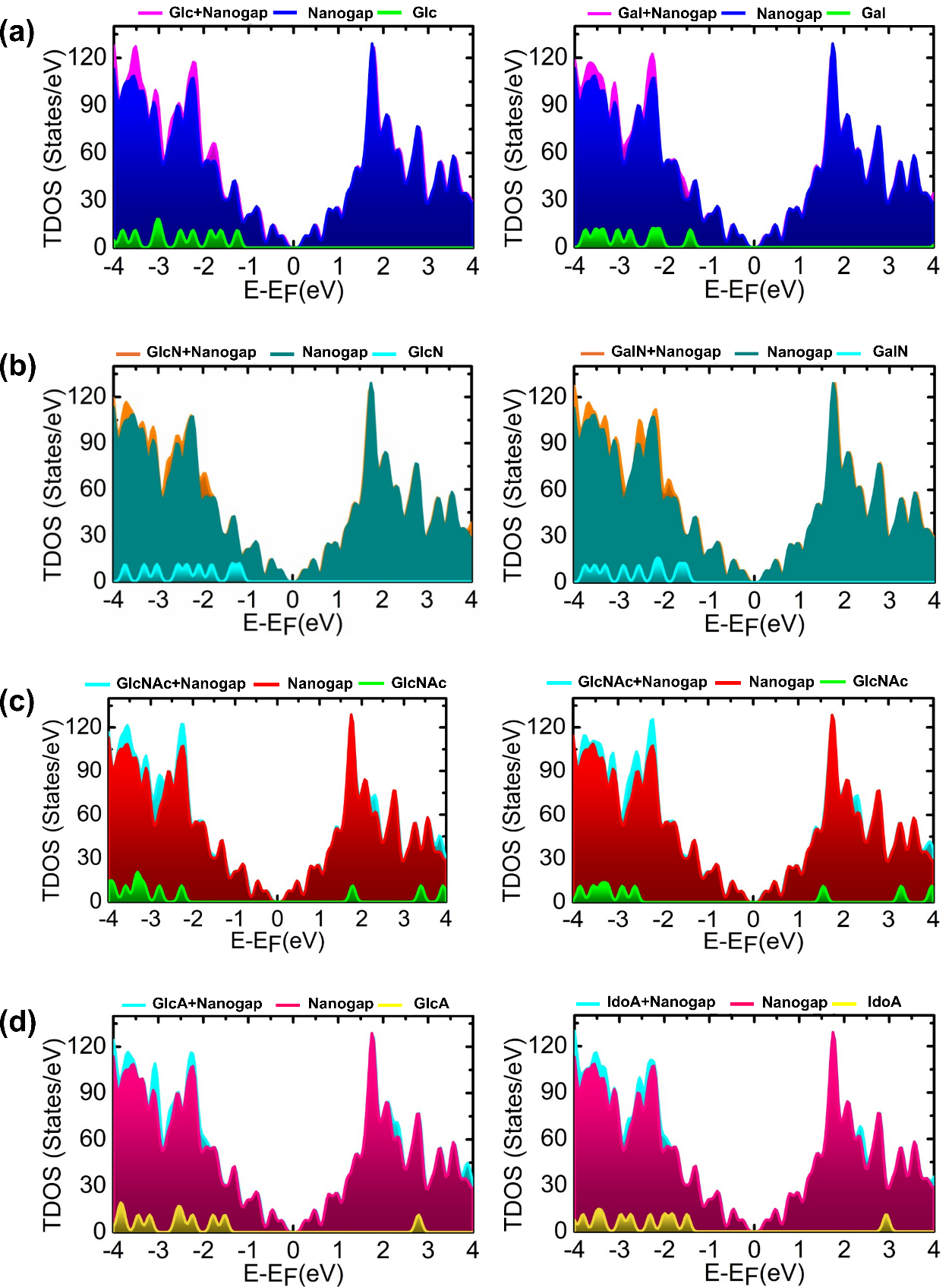


**Figure S6.** Total density of states (TDOS) plots of graphene-carbohydrate tunneling junction and the isolated nanogap and carbohydrate entities. **(a)** Glc and Gal, **(b)** GlcN and GalN, **(c)** GlcNAc and GalNAc, and **(d)** GlcA and IdoA. The Fermi energy level is shifted to zero.

**7. Transmission of Carbohydrate Stereoisomers with Molecular Orbital Wavefunctions**


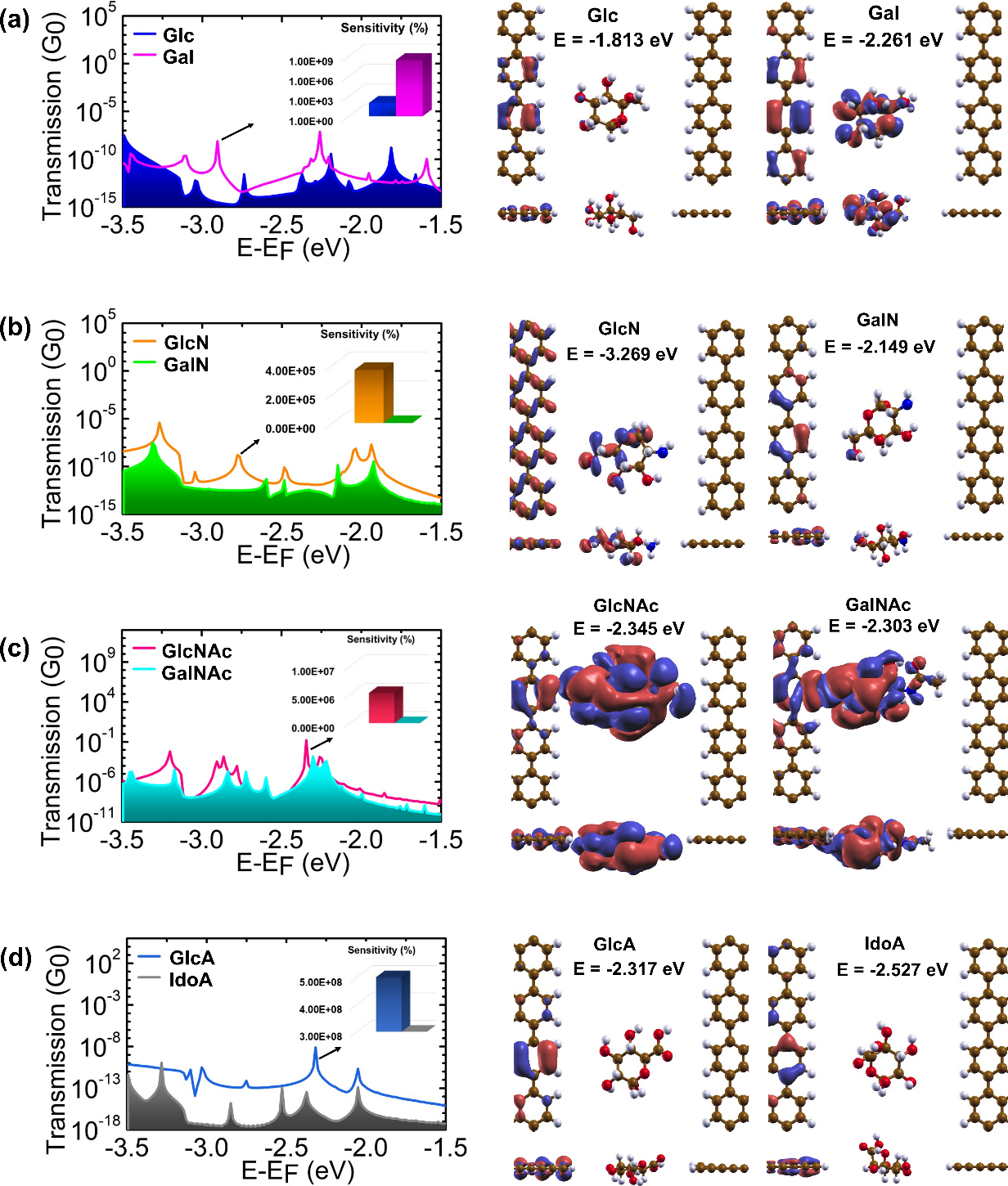


**Figure S7.** Zoomed zero-bias transmission energy profiles of carbohydrate stereoisomers and MOs isosurface plots (isosurface value is 0.01 e/$Å^{3}$) in their minimum energy configuration. **(a)** Glc and Gal, **(b)** GlcN and GalN, **(c)** GlcNAc and GalNAc, and **(d)** GlcA and IdoA. Inset shows the corresponding sensitivity (S%) = $\frac{G_{x}}{G_{0}}\times100$ bar plot at energies as marked by arrows, here $G_{x}$ and $G_{0}$ are the conductance of individual carbohydrate and reference carbohydrate (with lower transmission value), respectively. The Fermi energy level is set to zero. In the MOs wavefunction plots, the positive and negative lobes are shown in red and blue colors, respectively. Atom color code: C (brown), H (white), N (blue), and O (red).

**8. Molecular Fingerprints of Carbohydrate Stereoisomers with Physical Insights**


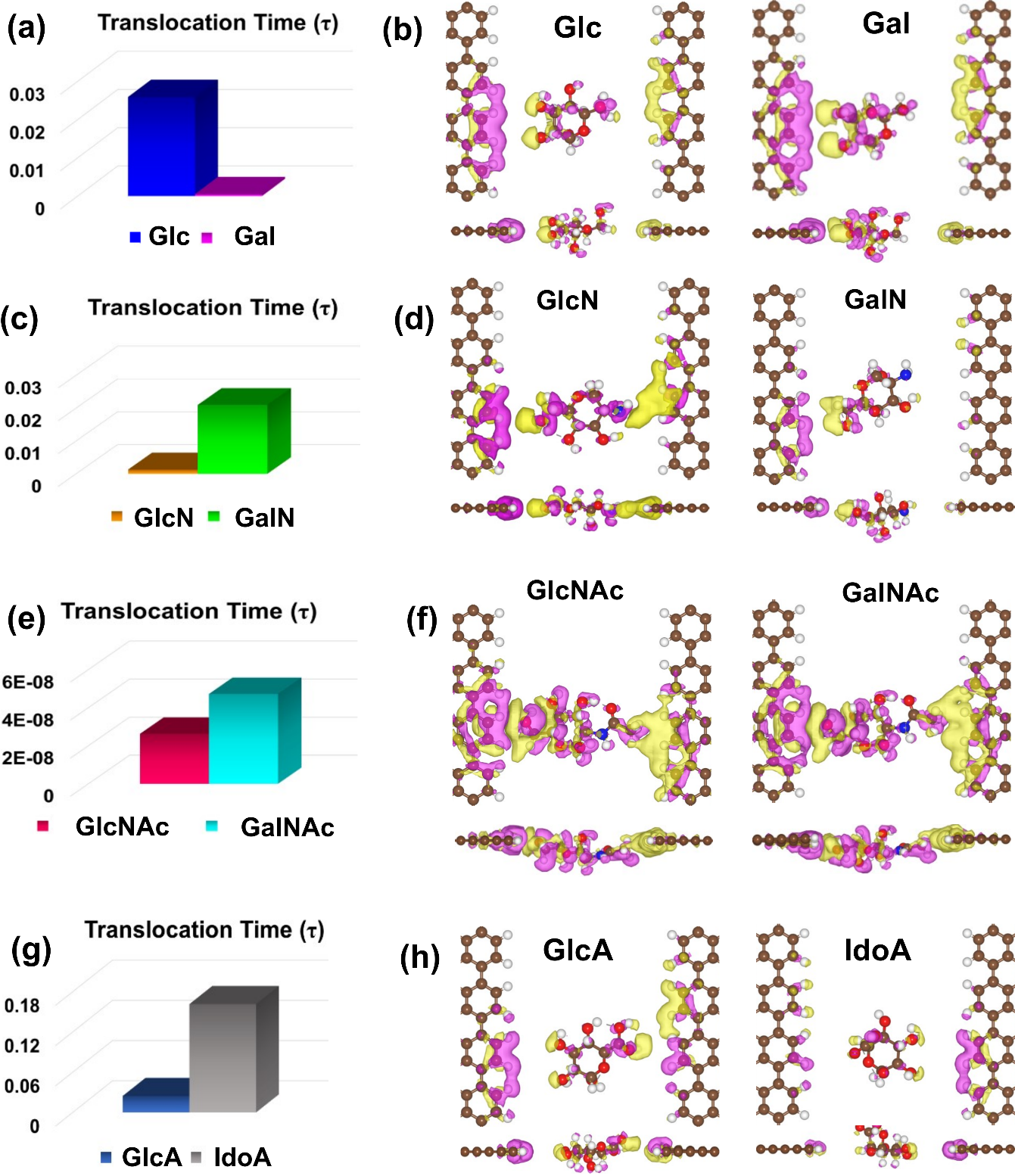


**Figure S8.** Translocation time ($\tau)$bar plots for carbohydrate stereoisomers and corresponding charge density difference (CDD) plots (isosurface value is 0.0005 e/$Å^{3}$). **(a)** Glc and Gal, **(b)** GlcN and GalN, **(c)** GlcNAc and GalNAc, and **(d)** GlcA and IdoA. In CDD Plots, the charge depletion and accumulation are represented by yellow and magenta colors, respectively. Atom color code: C (brown), H (white), N (blue), and O (red).

**9. Transmission profiles and Sensitivity with Different Rotation and Translation Dynamics**

**
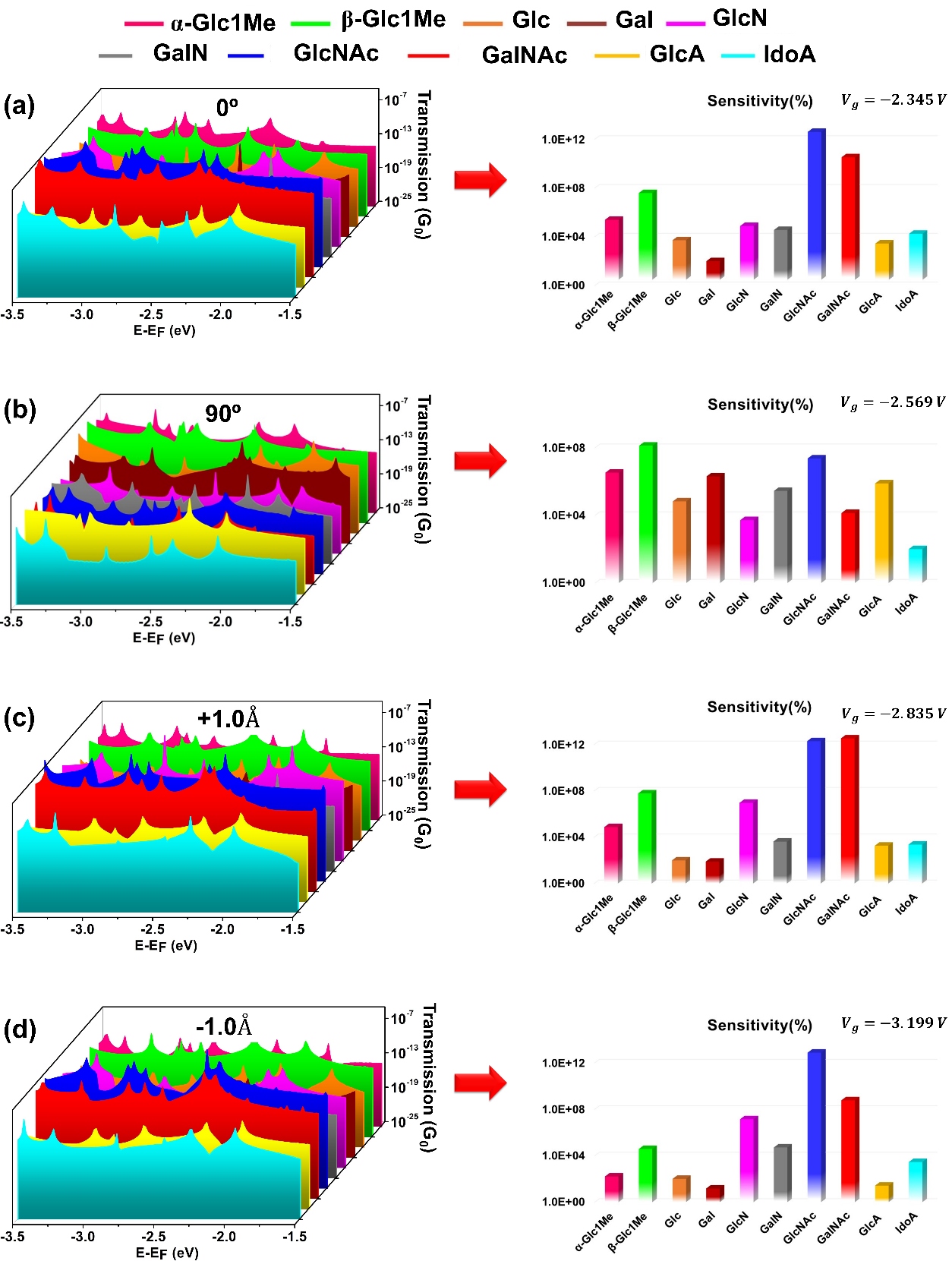
**

**Figure S9.** Combined signature transmission fingerprints of carbohydrate isomers and corresponding sensitivity histograms for individual sensing. **(a)** 0º orientation **(b)** 90º orientation, **(c)** +1.0 Å orientation, and **(d)** -1.0 Å orientation. The Fermi level is shifted to zero.

**10. Details of Tuned Hyperparameters**

**Table S1**. Tuned hyperparameters for selected ML classification algorithms.

| S. No. | Classification Models | Optimized Hyperparameters |
| --- | --- | --- |
| 1. | Logistic Regression (LR) | 'C': 0.01, 'class_weight': None, 'max_iter': 100, 'n_jobs': None, penalty= 'none', solver= 'newton-cg' |
| 2. | Random Forest Classification (RFC) | 'criterion': ‘gini,' 'max_depth’: 32, 'max_features': 'sqrt,' 'min_samples_leaf': 1, 'min_samples_split': 2, 'n_estimators': 100 |
| 3. | Decision Tree Classification (DTC) | 'criterion’: ‘entropy,' 'max_depth’: 5, 'max_features': 'None,' 'min_samples_leaf': 1, 'min_samples_split': 2, ‘min_weight_fraction_leaf’: 0.0 'random_state’: 38 |
| 4. | K-Nearest Neighbors Classification (KNC) | 'metric': 'manhattan,' 'n_neighbors': 1, ''weights': 'distance', 'algorithm': 'auto', 'leaf_size': 30, 'metric_params': None, 'n_jobs': None, 'n_neighbors': 5 |
| 5. | Support Vector Machines Classification (SVM) | 'C': 10.0, 'cache_size': 200, 'decision_function_shape': 'ovr', 'degree': 4, 'gamma': 'scale', 'kernel': 'rbf', 'max_iter': -1, 'shrinking': True, 'tol': 0.001 |
| 6. | Feedforward Neural Network (FNN) | ‘hidden layer’:1, ‘neurons’:10, ‘activation function’= ‘relu’, ‘epochs’: 100, ‘loss’: 'binary_crossentropy', optimizer='adam', batch_size=10, verbose=1 |

**11. Test and Validation Accuracy Scores for Optimized ML Algorithms**

**Table S2.** Computed accuracy scores for prediction of test dataset and validation (Val.) datasets with selected optimized ML classification algorithms.

|  | Accuracy (%) | | | | | | | | | |
| --- | --- | --- | --- | --- | --- | --- | --- | --- | --- | --- |
| ML Classification Algorithms | **α-Glc1Me**  **β-Glc1Me** | | **Glc**  **Gal** | | **GlcN**  **GalN** | | **GlcNAc**  **GalNAc** | | **GlcA**  **IdoA** | |
|  | **Test** | **Val.** | **Test** | **Val.** | **Test** | **Val.** | **Test** | **Val.** | **Test** | **Val.** |
| Logistic Regression (LR) | 51 | 48 | 48 | 47 | 43 | 54 | 58 | 51 | 42 | 60 |
| Random Forest Classification (RFC) | 100 | 99 | 99 | 95 | 97 | 99 | 99 | 100 | 99 | 100 |
| Decision Tree Classification (DTC) | 100 | 99 | 94 | 90 | 99 | 97 | 97 | 99 | 99 | 99 |
| K-Nearest Neighbors Classification (KNC) | 46 | 40 | 59 | 50 | 48 | 54 | 58 | 48 | 60 | 65 |
| Support Vector Machines (SVM) | 49 | 53 | 48 | 50 | 60 | 54 | 45 | 49 | 62 | 44 |
| Feedforward Neural Network (FNN) | 51 | 47 | 49 | 47 | 44 | 54 | 58 | 51 | 42 | 60 |

**12. RFC calling of Glc and Gal**


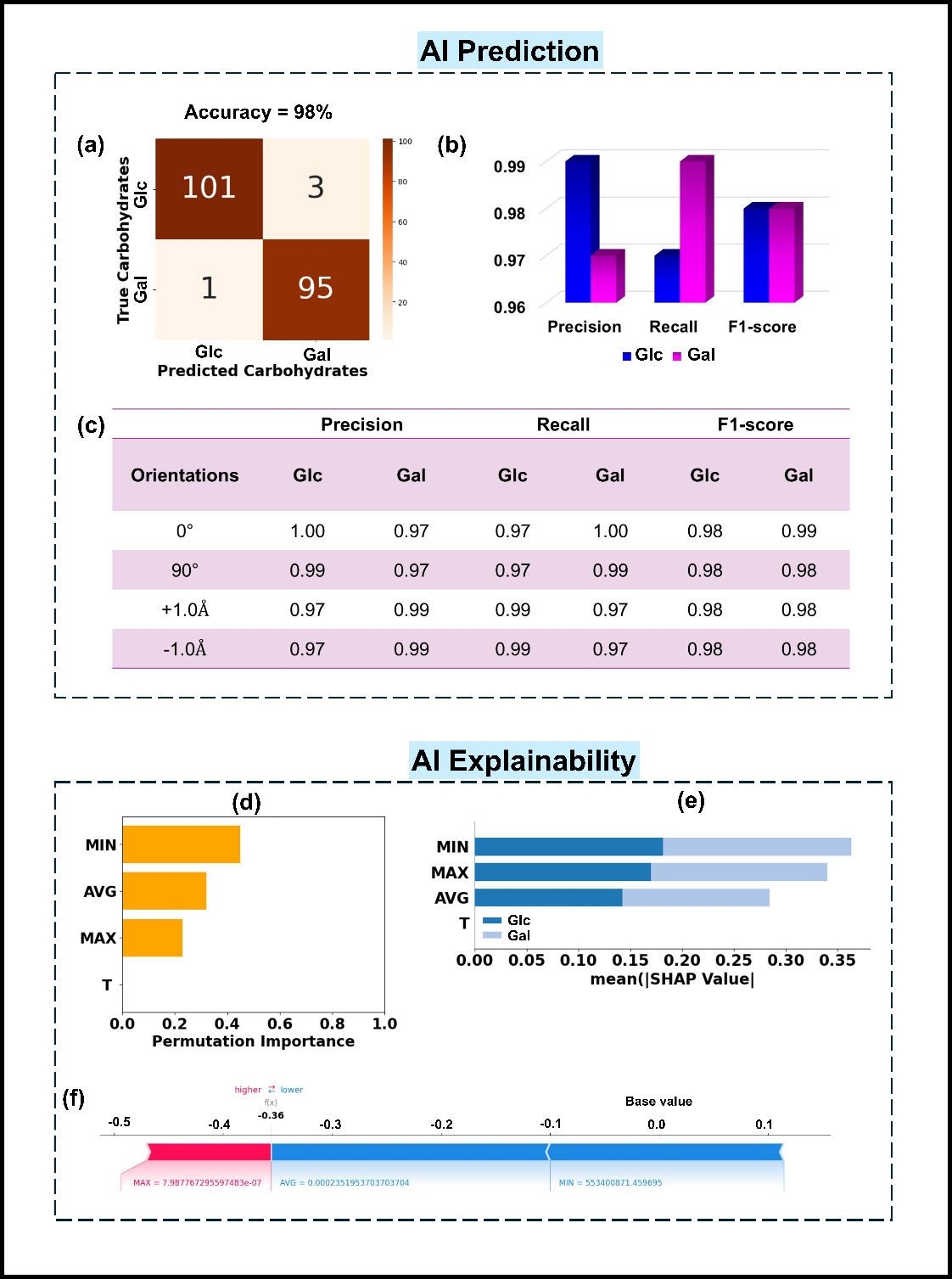


**Figure S10**. AI prediction of carbohydrate stereoisomer Glc and Gal with explainability. **(a)** confusion matrix for RFC prediction in their minimum energy configuration inside the tunneling junction, **(b)** classification report enclosing parameters precision, recall, and f1-score, **(c)** performance metrics for RFC prediction with different rotation and translation dynamics, **(d)** global feature importance plot, **(e)** SHAP summary bar plot illustrating contribution of each feature toward individual class, and **(f)** SHAP summary force plot illustrating contribution of features toward single prediction.

**13. RFC calling of GlcN and GalN**


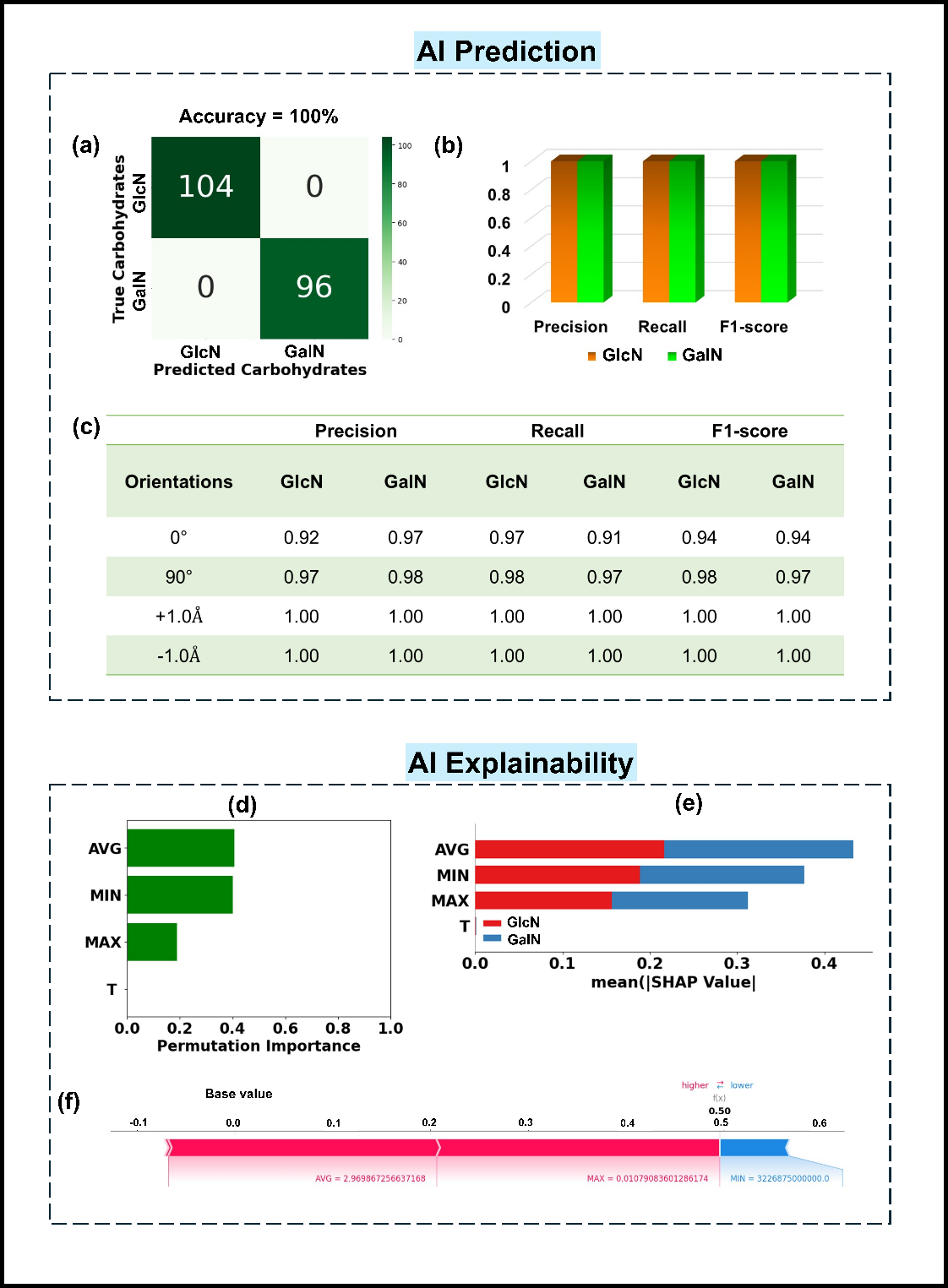


**Figure S11**. AI prediction of carbohydrate stereoisomer GlcN and GalN with explainability. **(a)** confusion matrix for RFC prediction in their minimum energy configuration inside the tunneling junction, **(b)** classification report enclosing parameters precision, recall, and f1-score, **(c)** performance metrics for RFC prediction with different rotation and translation dynamics, **(d)** global feature importance plot, **(e)** SHAP summary bar plot illustrating contribution of each feature toward individual class, and **(f)** SHAP summary force plot illustrating contribution of features toward single prediction.

**14. RFC calling of GlcNAc and GalNAc**


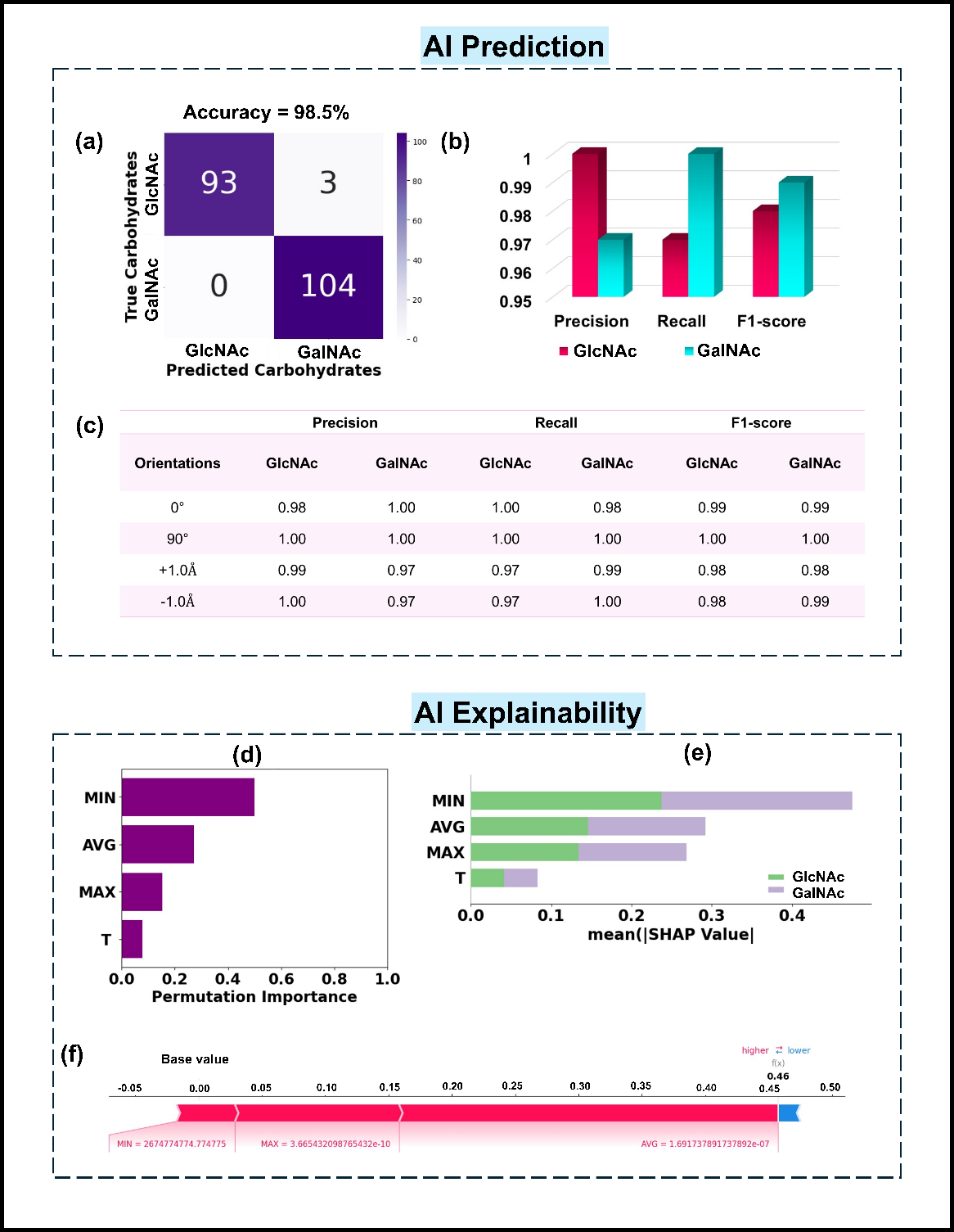


**Figure S12**. AI prediction of carbohydrate stereoisomer GlcNAc and GalNAc with explainability. **(a)** confusion matrix for RFC prediction in their minimum energy configuration inside the tunneling junction, **(b)** classification report enclosing parameters precision, recall, and f1-score, **(c)** performance metrics for RFC prediction with different rotation and translation dynamics, **(d)** global feature importance plot, **(e)** SHAP summary bar plot illustrating contribution of each feature toward individual class, and **(f)** SHAP summary force plot illustrating contribution of features toward single prediction.

**15. RFC calling of GlcA and IdoA**

**
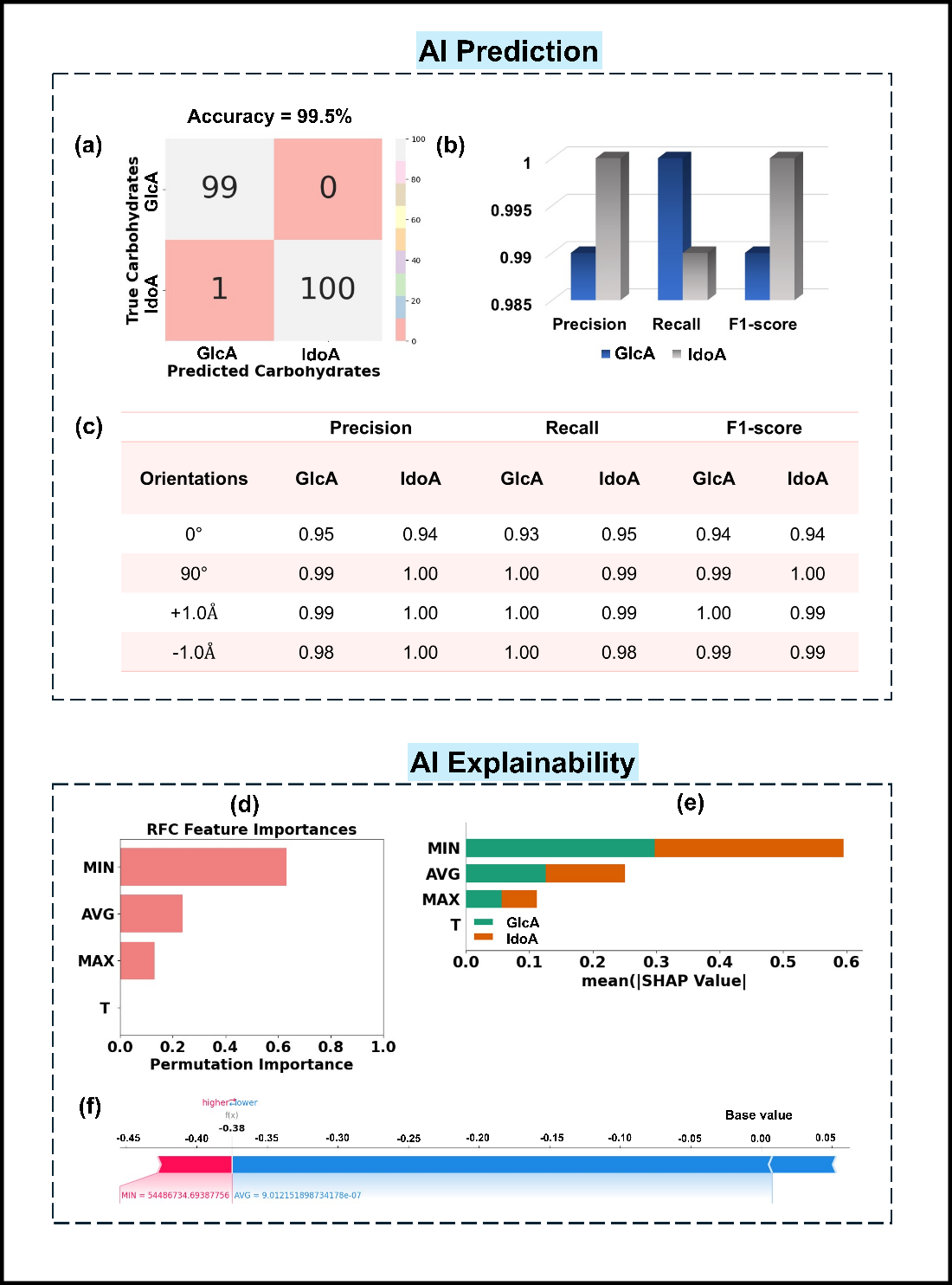
**

**Figure S13**. AI prediction of carbohydrate stereoisomer GlcA and IdoA with explainability. **(a)** confusion matrix for RFC prediction in their minimum energy configuration inside the tunneling junction, **(b)** classification report enclosing parameters precision, recall, and f1-score, **(c)** performance metrics for RFC prediction with different rotation and translation dynamics, **(d)** global feature importance plot, **(e)** SHAP summary bar plot illustrating contribution of each feature toward individual class, and **(f)** SHAP summary force plot illustrating contribution of features toward single prediction.

**16. RFC calling of All Ten Carbohydrate Isomers in 0º Orientation**


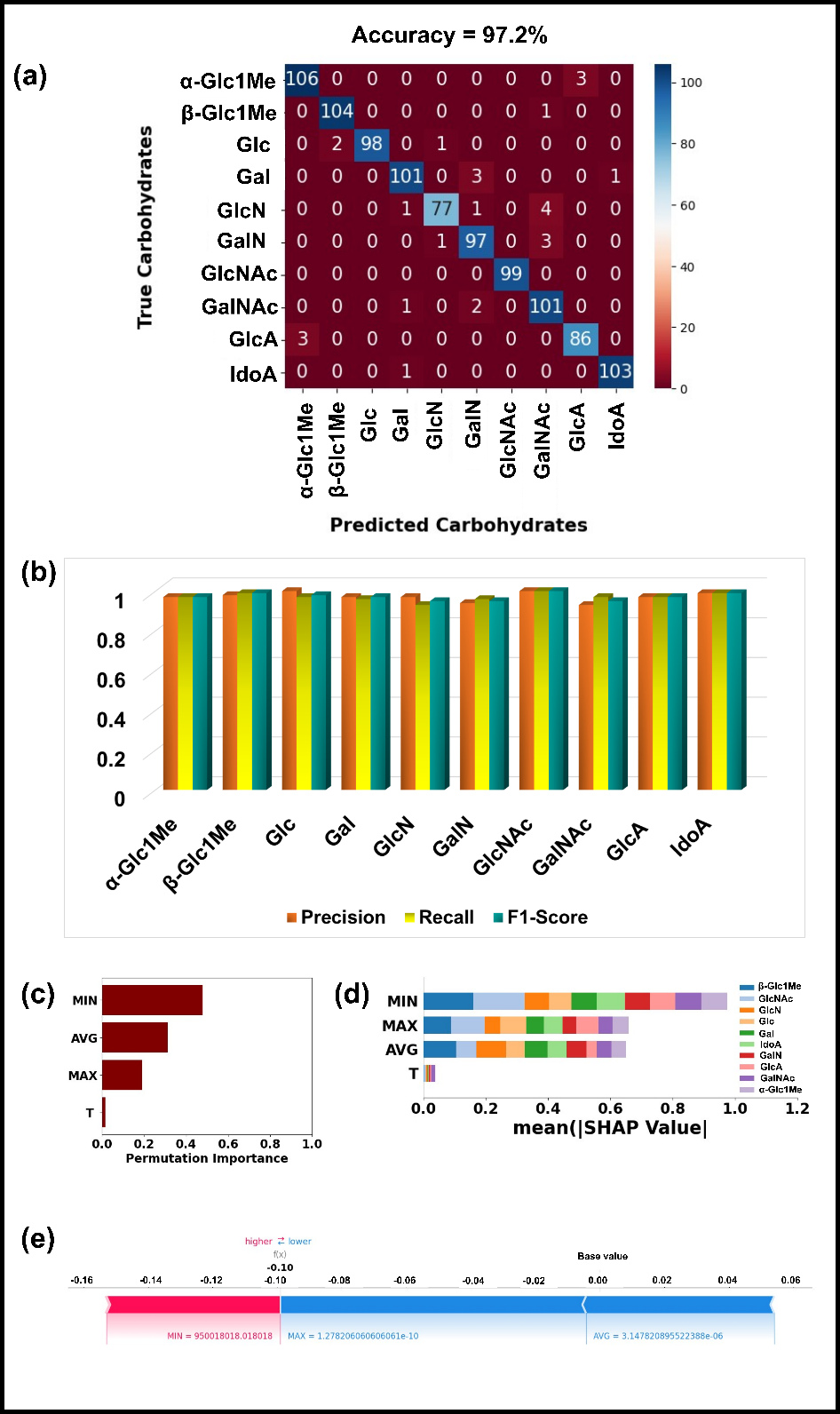


**Figure S14**. AI prediction of individual carbohydrates from a pool of QT transmission signals of all carbohydrate isomers in 0º Orientation. **(a)** confusion matrix, **(b)** classification report enclosing performance metrics precision, recall, and f1-score, **(c)** global feature importance plot, **(d)** SHAP summary bar plot illustrating contribution of each feature toward prediction of individual carbohydrate class, and **(e)** SHAP summary force plot illustrating contribution of features toward single prediction.

**17. RFC calling of All Ten Carbohydrate Isomers in 90º Orientation**


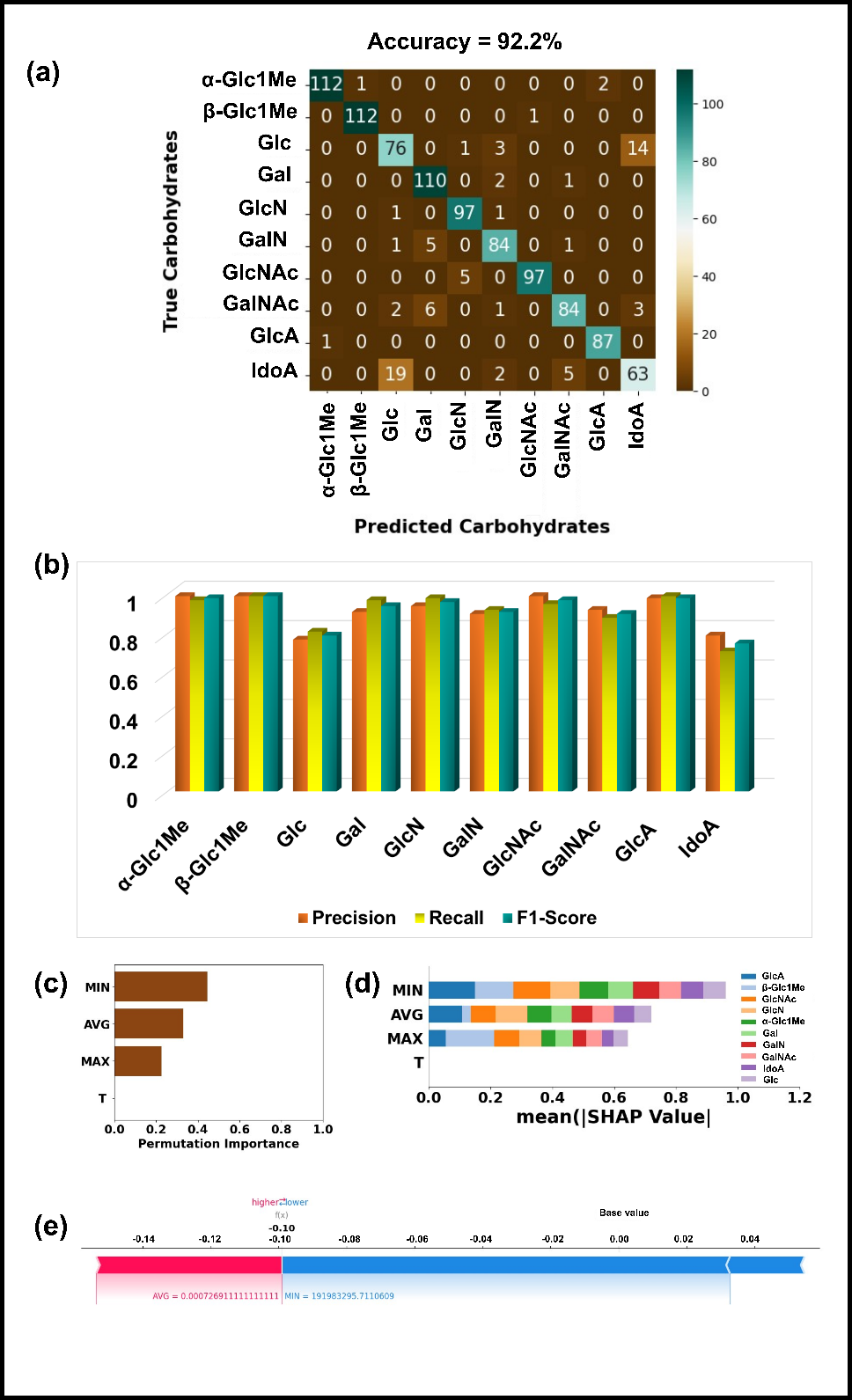


**Figure S15**. AI prediction of individual carbohydrates from a pool of QT transmission signals of all carbohydrate isomers in 90º Orientation. **(a)** confusion matrix, **(b)** classification report enclosing performance metrics precision, recall, and f1-score, **(c)** global feature importance plot, **(d)** SHAP summary bar plot illustrating contribution of each feature toward prediction of individual carbohydrate class, and **(e)** SHAP summary force plot illustrating contribution of features toward single prediction.

**18. RFC calling of All Ten Carbohydrate Isomers in +1.0 Å Orientation**


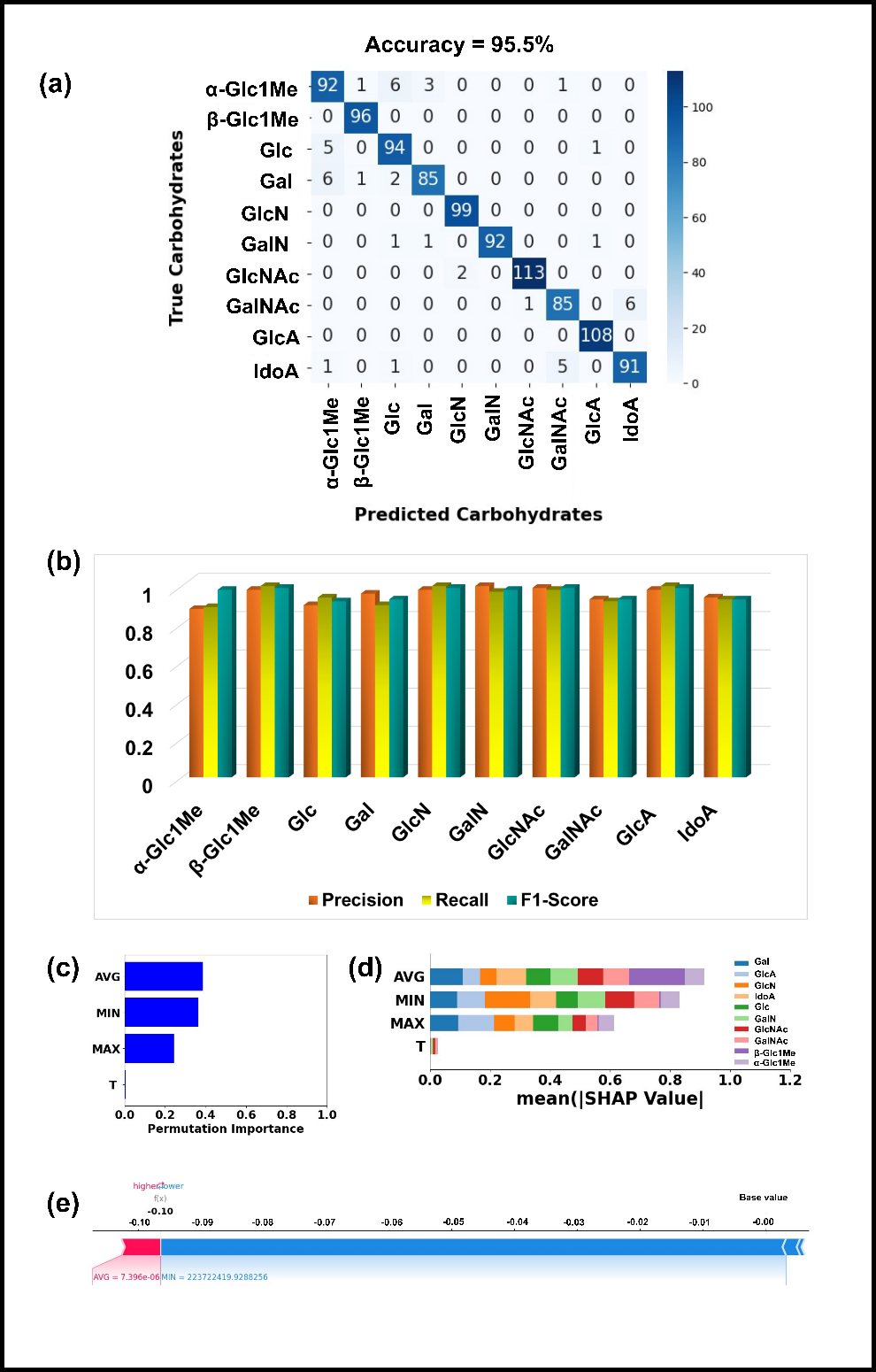


**Figure S16**. AI prediction of individual carbohydrates from a pool of QT transmission signals of all carbohydrate isomers in +1.0 Å Orientation. **(a)** confusion matrix, **(b)** classification report enclosing performance metrics precision, recall, and f1-score, **(c)** global feature importance plot, **(d)** SHAP summary bar plot illustrating contribution of each feature toward prediction of individual carbohydrate class, and **(e)** SHAP summary force plot illustrating contribution of features toward single prediction.

**19. RFC calling of All Ten Carbohydrate Isomers in -1.0 Å Orientation**


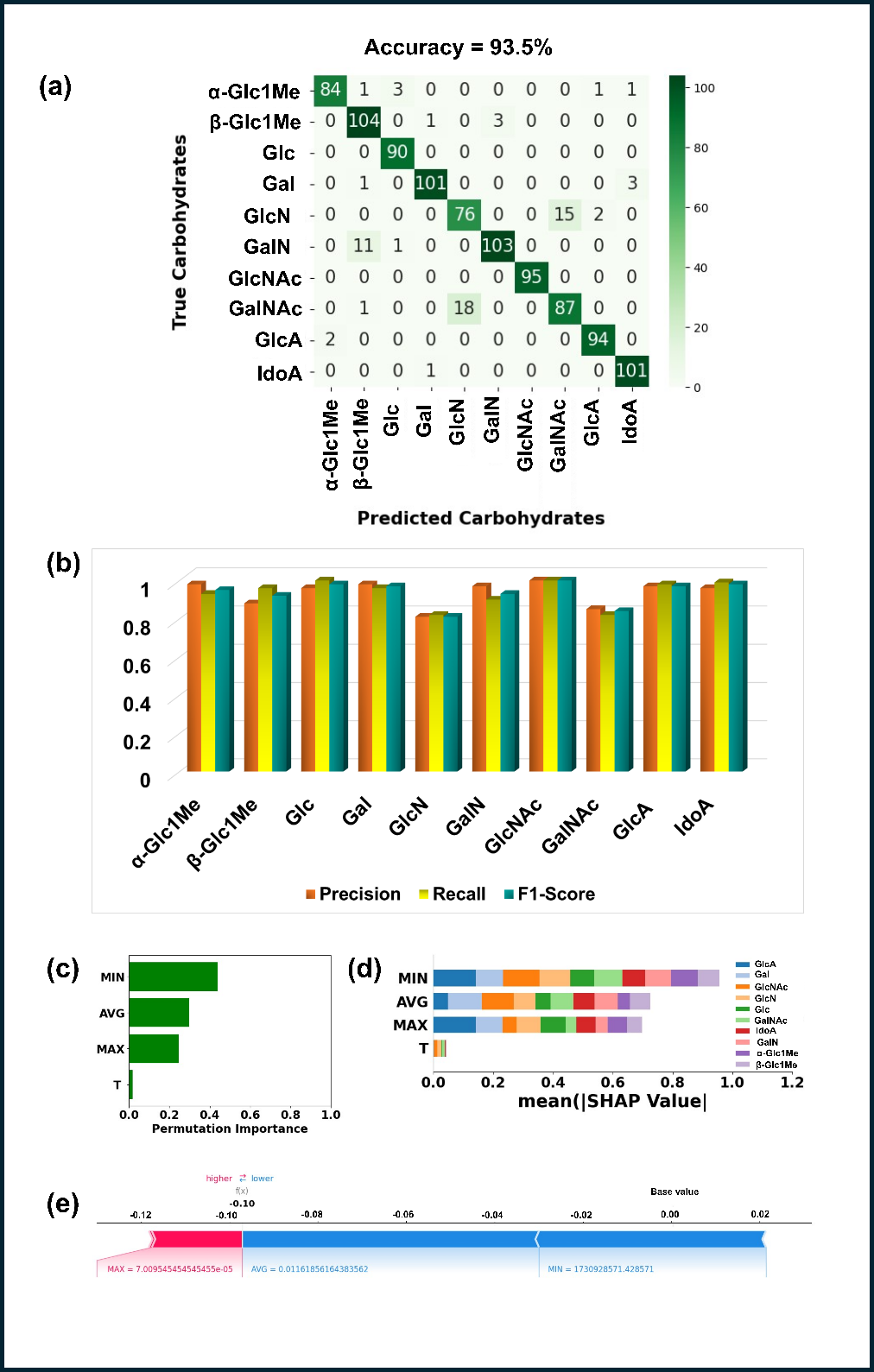


**Figure S17**. AI prediction of individual carbohydrates from a pool of QT transmission signals of all carbohydrate isomers in -1.0 Å Orientation. **(a)** confusion matrix, **(b)** classification report enclosing performance metrics precision, recall, and f1-score, **(c)** global feature importance plot, **(d)** SHAP summary bar plot illustrating contribution of each feature toward prediction of individual carbohydrate class, and **(e)** SHAP summary force plot illustrating contribution of features toward single prediction.

**20. 10-Fold Cross validation**

**Table S3.** 10-fold cross validation to check RFC stability in the prediction of individual monosaccharides with different rotation and translation dynamics.


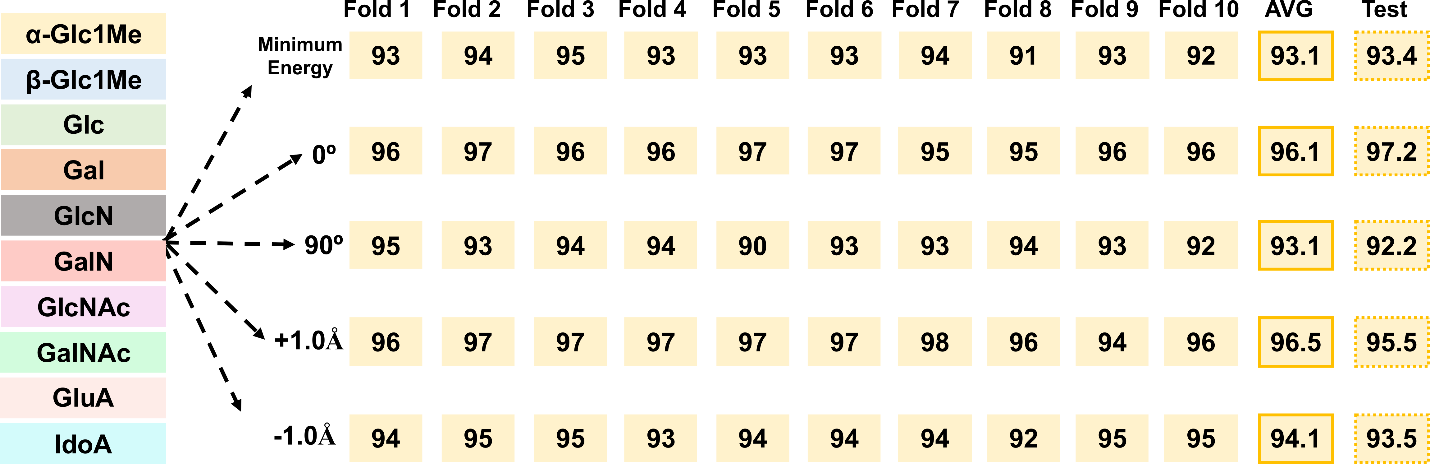


**21.** **Optimized Geometries of Carbohydrates**

**Table S4.** Optimized geometries and relative energy values (RE in Kcal/mol) of considered monosaccharides using different level of theories B3LYP/6-31+G*, wb97xd/def2tzvp, and PBE0+D3/6-311+G(d,p).

**
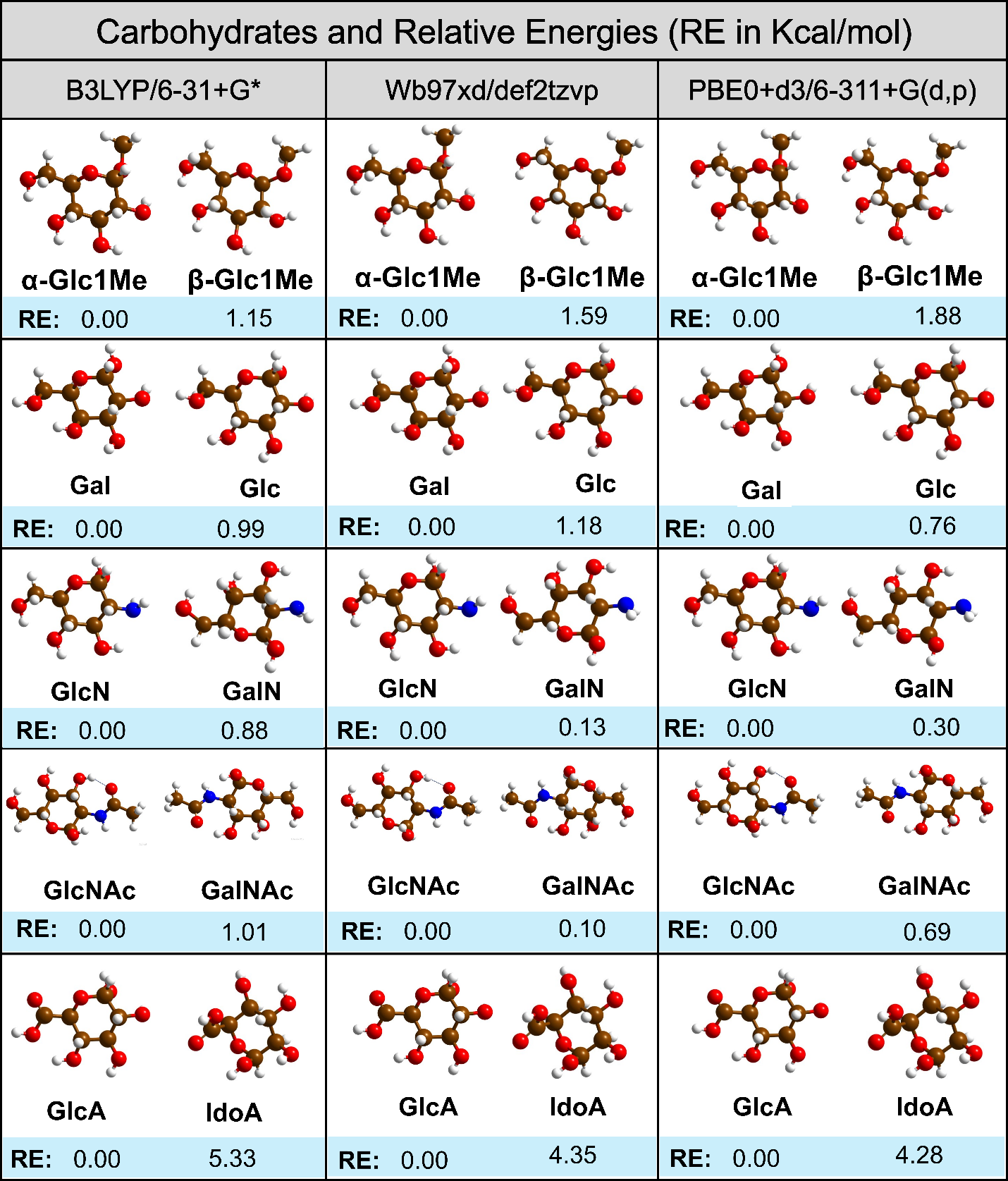
**

**21.** **Optimized Geometries of Graphene-Carbohydrate-Graphene Systems**

**Table S5.** Optimized geometries and relative energy values (RE in eV) of considered graphene-carbohydrate-graphene systems using different two different levels of theories, vdW-DZP and vdW-TZP.

*
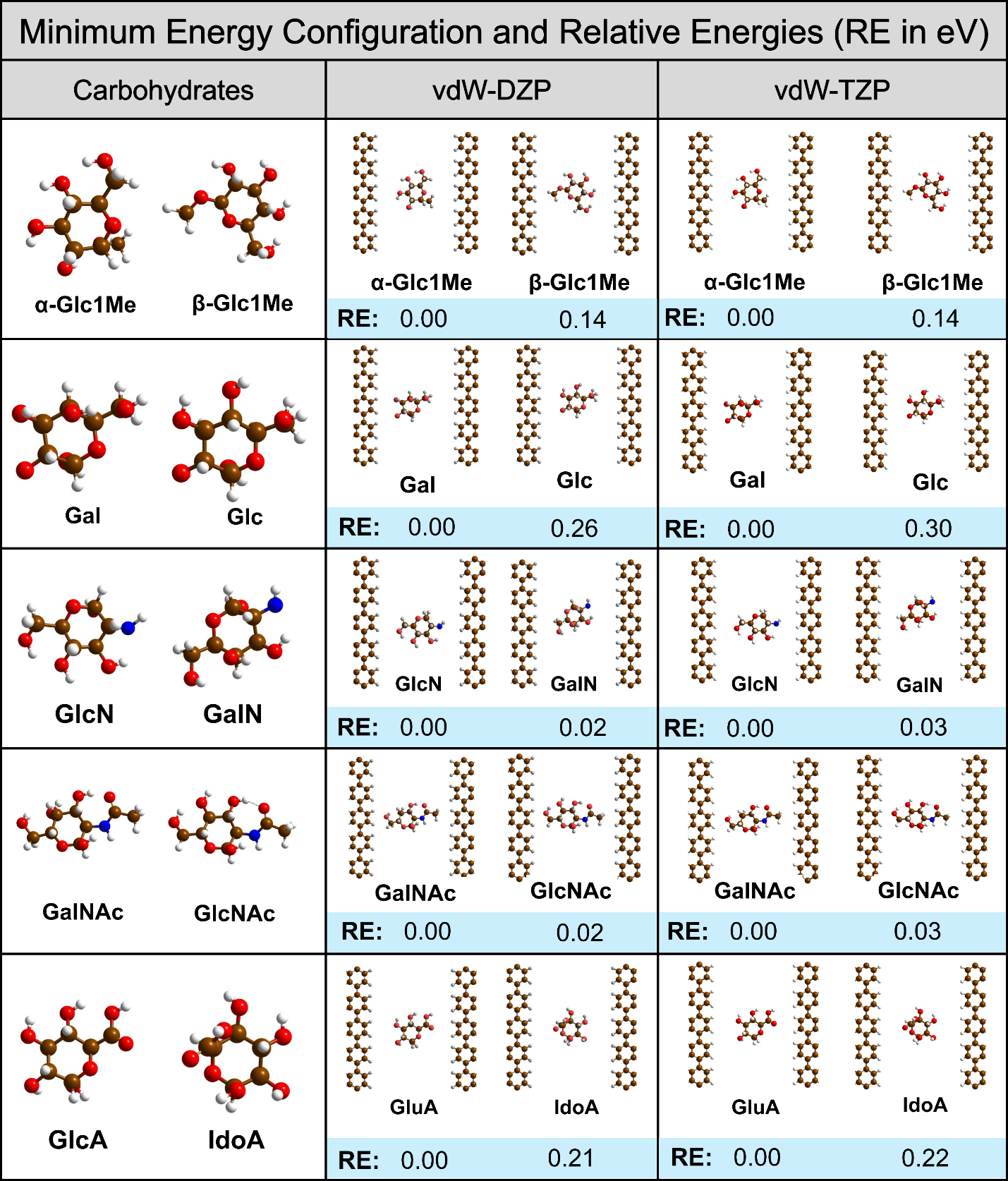
*
